# Influence of CO_2_-Mineralised Alkaline Waste Materials on the Chloride Ingress Resistance of Sustainable Cementitious Materials

**DOI:** 10.3390/ma19204353

**Published:** 2026-10-16

**Authors:** María Victoria Paredes, Ana Guerrero, Ana María Moreno de los Reyes, Javier Sánchez, Julio Torres, Jaime Moreno-Juez, Moisés Frías, Milica Vidak Vasić

**Affiliations:** 1 Eduardo Torroja Institute for Construction Sciences, Spanish National Research Council (IETcc-CSIC), 28033 Madrid, Spain; ana.moreno@ietcc.csic.es (A.M.M.d.l.R.); javier.sanchez@csic.es (J.S.); juliotorres@ietcc.csic.es (J.T.); mfrias@ietcc.csic.es (M.F.); 2 Tecnalia, Basque Research and Technology Alliance (BRTA), Astondo Bidea, ED. 700, Parque Tecnológico de Bizkaia, 48160 Derio, Spain; jaime.moreno@tecnalia.com; 3 Institute for Testing of Materials IMS, 11040 Belgrade, Serbia; milica.vasic@institutims.rs

**Keywords:** carbonated alkaline waste materials, chloride resistance, eco-mortars, durability, supplementary cementitious materials

## Abstract

**Highlights:**

- CO_2_-mineralised alkaline wastes were evaluated as supplementary cementitious materials in blended cements.
- The influence of waste pre-carbonation on chloride ingress resistance depended on waste type and replacement level.
- Mineralogical changes were monitored using FTIR, DTG/TG, and XRD.
- Mortars with carbonated waste exhibit lower chloride diffusion coefficients than their non-carbonated counterparts.
- Pre-carbonation may enhance the suitability of selected wastes for cementitious applications.

**Abstract:**

Using CO_2_-mineralised alkaline waste as a supplementary cementitious material has the potential to advance sustainable construction practices. This paper evaluates the effect of carbonating three fine industrial waste materials—construction and demolition waste, ladle furnace slag, and biomass ash—on the durability performance of blended cements in which they replace 7% and 20% of ordinary Portland cement. The performance of carbonated waste was compared with that of their non-carbonated counterparts through chloride ingress resistance, a key parameter governing reinforcement corrosion. The results show that prior carbonation can significantly influence chloride transport properties, although the magnitude and direction of the effect depend strongly on the waste type and replacement level. Mortars with carbonated construction and demolition waste or ladle furnace slag generally reduced the chloride diffusion coefficient compared with the corresponding non-carbonated materials. This behaviour was associated with greater portlandite consumption and enhanced chloride-binding capacity. Nevertheless, none of the blended systems outperformed the ordinary Portland cement (OPC ) in terms of chloride diffusion resistance. Conversely, biomass ash at 20% replacement led to markedly reduced performance, with the estimated time to steel depassivation decreasing to 1–2 years under the exposure conditions considered. These findings indicate that prior carbonation may improve the suitability of some wastes for cementitious applications relative to their non-carbonated counterparts, although the resulting chloride resistance remains lower than that of OPC. The benefits are of waste carbonation are therefore material-dependent and should be assessed on a case-by-case basis.

## 1. Introduction

Corrosion of the steel reinforcements in reinforced concrete structures is one of the primary deterioration mechanisms affecting the durability of infrastructure exposed to the aggressive environments found in coastal, industrial and urban areas. This electrochemical phenomenon is triggered when external agents (e.g., chloride ions or carbon dioxide) penetrate the concrete cover, disrupt the protective alkaline environment surrounding the steel and initiate oxidation.

The consequences include cracking, spalling of the concrete cover, loss of cross-sectional area in the steel reinforcements, and, in extreme cases, structural failure. There is no consensus in the literature on the chloride concentration threshold that triggers concrete degradation, though values such as Cl^−^/OH^−^ = 0.6 [1] or 0.6% chloride by weight of cement [2,3] are frequently cited. Numerous studies have used theoretical, numerical or experimental approaches to model these processes [4,5,6,7].

Chloride ions penetrating the concrete’s pore network can be partly bound to the hydrated phases—primarily C-S-H gel—and/or react chemically with C_3_A and C_4_AF to form Friedel’s salt (Ca_4_Al_2_(OH)_12_Cl_2_∙4H_2_O) and, to a lesser extent, react with C_3_S (whose slower hydration limits its contribution) [8,9]. Depending on the chloride concentration (< or > 1.0 mol∙L^−1^), either physical adsorption or chemical reaction predominates [10,11]. Chloride-binding capacity is known to depend on multiple factors, including cement type, the nature of the cation associated with the salt, chloride content, water/binder ratio, exposure time, curing temperature, Ca/Si ratio, pH and hydroxide concentration [12,13,14,15,16,17].

It is the free chlorides present in the concrete’s pore network, however, that are potentially responsible for initiating and propagating corrosion in the embedded steel reinforcements and, ultimately, for degrading concrete structures [13].

In parallel, growing concern over the cement industry’s environmental impact has driven development of more sustainable and durable materials [18,19,20]. The incorporation of supplementary cementitious materials (SCMs) such as ground granulated blast-furnace slag and fly ash not only enhances concrete’s resistance to aggressive agents but also significantly reduces CO_2_ emissions associated with Portland cement production. Recent studies highlight the importance of integrating durability and sustainability criteria into the design and maintenance of infrastructure, thus encouraging the construction sector’s transition toward a circular economy [21,22,23]. To this end, highly reactive pozzolans such as metakaolin (MK), which supply reactive silica and alumina, are used to enhance resistance to chloride ion diffusion/migration [24,25]. Similar behaviour is observed in cement-based matrices incorporating other non-traditional industrial waste materials—red mud, bauxite tailings [26], Ichu grass ash [27], coal mining waste [28] and recycled construction and demolition waste (CDW). In the latter case, the nature of the waste material (concrete or brick powder) determines chloride ion penetration resistance [29], with concrete CDW-based matrices performing better [30,31]. This aligns with the findings of previous studies [32] analysing different recycled concrete powders (siliceous and calcareous) in ternary cements, which found that calcareous concrete CDW lowered resistance to chloride ion attack. Cement, long regarded as an emissions-intensive material, is thus emerging as an active ally in the fight against climate change.

The shift towards sustainable construction is profoundly transforming development of cement-based materials. Within this framework, current research is focusing on emerging CO_2_ sequestration technologies applicable to cement matrices in both fresh and hardened states. These solutions not only represent a technical advance but are also emerging as key strategies in the construction sector’s roadmap to decarbonisation and to achieving its 2050 climate neutrality targets [33,34,35,36,37,38,39,40,41].

Against this backdrop, a new approach is gaining traction: use of alkaline waste materials as CO_2_ sinks prior to employing them as SCMs. This strategy transforms industrial waste into functional resources, integrating carbon capture and circular economy principles into the design of next-generation construction materials. Despite the significant scientific and technical gaps that remain in this research field, early studies have shown that mineralising alkaline waste materials (such as fly ash, steel slag and fine-fraction CDW) by exposing them to CO_2_ promotes the formation of calcite as the dominant phase because of its high stability. Furthermore, mineralisation generates silica and alumina gels as a result of decomposition of the hydrated phases (C-S-H gels and aluminous compounds), rendering these waste materials more reactive and stable after carbonation. This transformation is particularly relevant in cement-based matrices containing potentially expansive elements and/or compounds, such as steel slags, biomass ashes or CDW, where it enhances both durability and mechanical performance [42,43,44,45]. However, while previous studies have mainly focused on CO_2_ sequestration efficiency, mineralogical transformations and mechanical performance, the influence of prior carbonation on chloride-related durability when these carbonated wastes are subsequently used as SCMs remains insufficiently understood. In particular, direct comparisons between carbonated and non-carbonated waste-based systems, as well as assessment of their performance relative to OPC under chloride exposure conditions, are still scarce.

To address this knowledge gap, this paper aims to advance the understanding of how eco-cement mortars made with previously carbonated alkaline waste materials (7% and 20% replacement content) behave under non-steady-state chloride penetration. To this end, chloride ion concentration profiles are analysed and the service life of the cement matrix prior to corrosion onset is estimated. By comparing carbonated and non-carbonated formulations and benchmarking this behaviour against OPC, this study provides new insights into the effect of prior CO_2_ mineralisation on chloride transport and durability-related parameters. The results contribute to a better understanding of the potential benefits and limitations associated with the use of carbonated alkaline waste materials as eco-SCMs in chloride rich environments.

## 2. Materials and Methods

### 2.1. Starting Materials

For this study, three Spanish industrial waste materials were selected that, owing to their characteristics, are not currently used in commercial cement manufacture: (1) a fine fraction of siliceous concrete construction and demolition waste (CDW-C) with a particle size of <5 mm generated during the crushing and screening process employed to produce the various grades of commercial recycled coarse aggregate, stockpiled in the open at the waste management plants themselves and accounting for up to 40% of processed CDW volume; (2) a white ladle furnace steel slag (LFS) with a particle size of <4 mm; and (3) a biomass ash (BA) derived from cereal and forest waste and produced by a company that uses biomass as an alternative fuel. All the waste materials were ground to <45 µm to achieve a fineness comparable to ordinary Portland cement (OPC). The chemical compositions determined by X-ray fluorescence (XRF) are summarised in Table 1. All quantified oxides are reported, while those present at concentrations below 5 wt.% have been grouped under the category “minor oxides” (Fe_2_O_3_, Na_2_O, TiO_2_, P_2_O_5_) to simplify the presentation of the results. Further details on these analyses can be found in previous papers [30,46].

LFS was mainly composed of CaO (48.81 wt.%) and MgO (11.66 wt.%), while CDW-C exhibited a predominantly siliceous composition, with SiO_2_ accounting for 43.81 wt.%. BA showed a distinct chemical composition characterised by high CaO, SO_3_ and K_2_O contents.

The mineralogical composition determined by X-ray diffraction (XRD) and quantified using the–Rietveld refinement method is presented in Table 2. The identified crystalline phases are in good agreement with the chemical compositions obtained by XRF analysis. LFS exhibited a predominantly calcium- and magnesium-rich composition, which is reflected in the presence of periclase (8%), Ca-olivine (10%), portlandite (9%), hydrogarnet (7%) and gibbsite (6%). The high CaO (48.81 wt.%) and MgO (11.66 wt.%) contents determined by XRF are therefore associated with these calcium- and magnesium-bearing phases. In addition, the high amorphous fraction (60%) suggests the presence of a significant vitreous component, which could contribute to the reactivity of the material.

BA showed a more complex mineralogical assemblage consisting of periclase (10%), katoite (10%), calcite (12%), dolomite (11%), portlandite (13%) and potassium sulphate (13%). This mineralogical composition is consistent with the elevated CaO, SO_3_ and K_2_O contents measured by XRF. In particular, the presence of K_2_SO_4_ explains the high concentrations of both potassium (17.53 wt.% K_2_O) and sulphur (10.41 wt.% SO_3_), while calcite and dolomite contribute to the CaO content and are consistent with the high LOI value (21.34 wt.%), which is likely associated with carbonate decomposition during ignition. CDW-C was characterised by a predominantly siliceous mineralogy, with quartz representing 22% of the crystalline phases, accompanied by calcite (17%), mica (8%), plagioclase (9%) and K-feldspar (6%). These results are in agreement with the XRF analysis, which showed the highest SiO_2_ content among the investigated materials (43.81 wt.%). The significant calcite content further explains the relatively high CaO concentration and loss on ignition (LOI) value, indicating the presence of carbonated cementitious and mineral fractions typically associated with construction and demolition waste.

A commercial CEM I 52.5R ordinary Portland cement (OPC) complying with European standard EN 197-1 [47] was selected as the reference for the manufacture of low-carbon eco-cements. Binary blended cements were then prepared by replacing 7% and 20% of the OPC with each of the carbonated and non-carbonated waste materials. These replacement levels were chosen because they fall within the substitution range typically associated with CEM II-A-type formulations, although the resulting blends are not intended to be classified as CEM II/A cements, since a 20% replacement would reduce the clinker content below the minimum 80% required by the standard. The chemical (XRF), physical (Brunauer–Emmett–Teller theory BET-N_2_), mineralogical (XRD–Rietveld) compositions of the anhydrous blended cements and mechanical properties are described in previous papers [48,49].

### 2.2. Blended Cement Mortars

Thirteen mortars were prepared in accordance with European standard EN 196-1 [35]: 1 OPC reference mortar, 6 containing carbonated waste materials and 6 containing non-carbonated waste materials. The standard sand used had a silica content of ≥98%, the binder/sand ratio was 1:3 and the water/binder ratio was 0.5. The mortars were cured for 28 d prior to chloride testing and subsequent characterisation.

### 2.3. Test Methodology

#### 2.3.1. Carbonation of the Starting Materials

The alkaline waste materials were carbonated at Tecnalia’s facilities in Bilbao using a 5 L static reactor under the following conditions: (a) relative humidity: 10% for the CDW-C and 15% for the LFS and 30% for the BA; (b) commercial dry 100% CO_2_ at a flow rate of 2 L∙min^−1^ for 90 min under standard conditions of 20 °C and 1 atm (longer exposure times yielded negligible additional carbonate formation). Calcimetric analysis showed CO_2_ uptake of 8.0, 42.7 and 60.5 eq CO_2_/kg for the CDW-C, LFS and BA, respectively. The carbonated waste materials, carbonated concrete construction and demolition waste (CCDW-C), carbonated white ladle furnace steel slag (CLFS), carbonated biomass ash (CBA) were then dried in an oven at 105 °C for 24 h and ground to <45 µm.

The chemical compositions determined by X-ray fluorescence (XRF), the mineralogical compositions determined by X-ray diffraction (XRD–Rietveld) and Dx and BET-N_2_ values for carbonate wastes can be found in previous papers, together with other physical properties [48,49].

#### 2.3.2. Non-Steady-State NaCl Ponding Test

**Figure 1:**
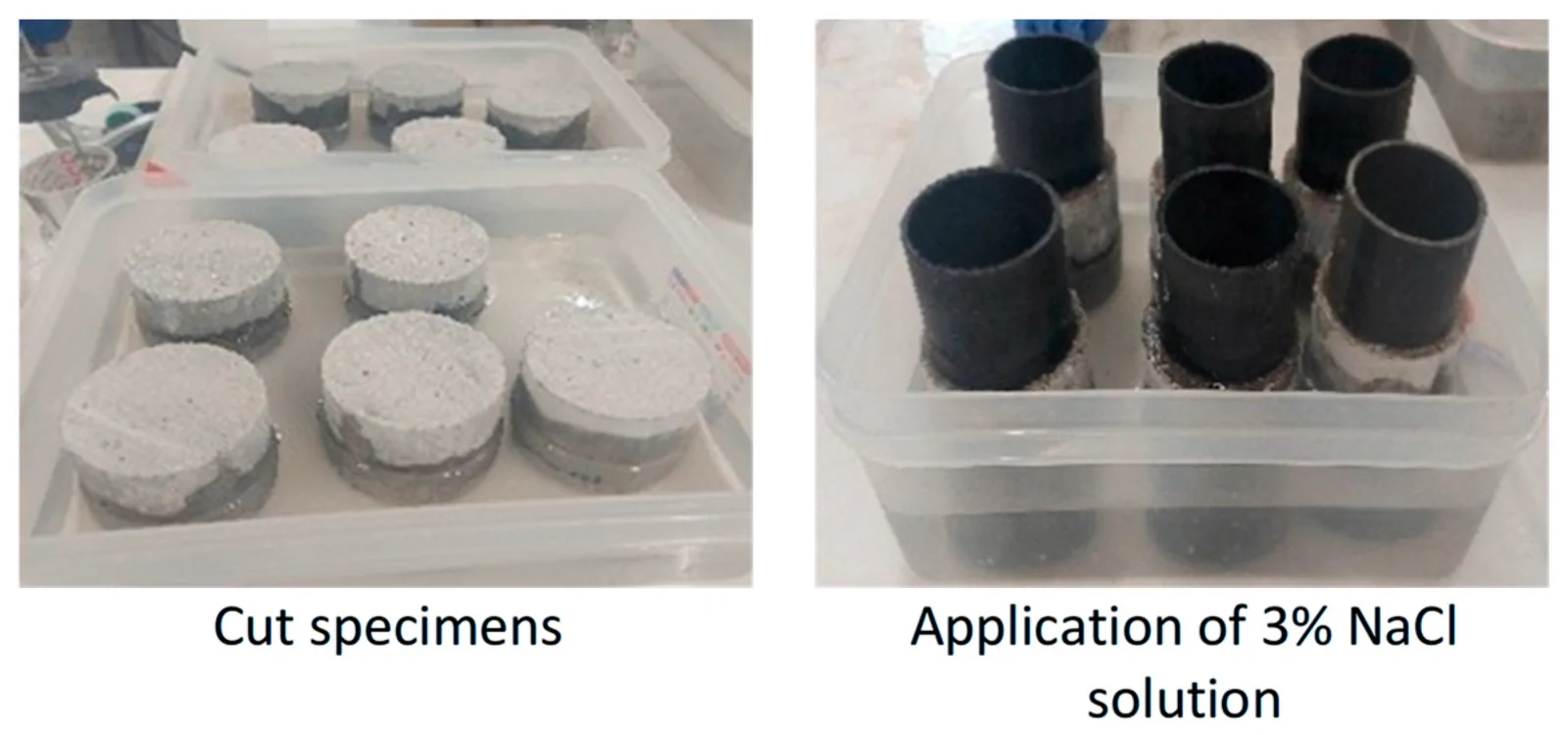
Specimen appearance (***left***) and 3% NaCl ponding test on the cut surface (***right***).

Chloride testing was performed in accordance with European standard UNE EN 12390-11 [50]. Cylindrical specimens (ø7.5 × 15 cm) were cured and conditioned then cut in half to produce ø7.5 × 7.5 cm half-specimens, with two specimens per formulation. It is at this stage that the mortars are briefly exposed to atmospheric CO_2_, enabling its capture and interaction (it is in this step that the mortars are exposed to atmospheric CO_2_ for a short time, making it possible to capture and interact with them).The ponding test involves first placing a ring (made of acrylic or polyvinyl chloride, (PVC) over the exposed surface to contain the saline solution and sealing its outer edges with epoxy resin to ensure unidirectional flow from the top to the bottom. A 3% NaCl solution is then applied to the upper surface and kept sealed for 90 days of exposure (Figure 1).

After the exposure period, the solution was removed by cleaning the surface, and dust was extracted from each mortar (two independent specimens were tested for each mortar formulation) by drilling in layers at different depths in accordance with the European standard UNE EN 12390-11 for a water-to-cement ratio of 0.5 [50].

#### 2.3.3. Chloride Quantification and Diffusion Modelling

Powder samples were collected at the specified depths from each specimen, and after drying the powders at 40 °C for 24 h, total chloride content was quantified by XRF in triplicate at each depth to ensure an error of <5% [2,51]. The detailed calibration, matrix-effect assessment and validation of the portable XRF procedure against acid-soluble end-point potentiometric titration have been reported previously [2,51]. In that validation, comparison of the XRF and potentiometric results established a correction factor of 1.16 for chloride quantification [2]. The chloride concentration was determined using an Olympus X-Innov-X 2000 portable XRF analyser, with the quantification results expressed in parts per million (1 ppm = 0.0001%). The chloride profiles were fitted to Fick’s second law by non-linear least-squares regression (Equation (1)):

(1) $$C_{x,t}=C_{0}\left[1-\mathrm{erf}\left(\frac{x}{2\sqrt{\mathrm{Dt}}}\right)\right]$$

where *Cx,t*, is the chloride concentration (%) at each depth at time t (s), *C*_0_ is the calculated chloride concentration (%) at the mortar surface exposed to the solution, and *D* the non-steady-state diffusion coefficient (cm^2^∙s^−1^).

Non-linear regression using the least-squares method was employed to determine the chloride content at the exposed mortar surface (*C*_0_). The uncertainties reported for the fitted *Co* and D values correspond to the uncertainties derived from the non-linear regression and should not be interpreted as specimen-to-specimen variability. 

### 2.4. Instrumental Techniques

Fourier transform infrared (FTIR) analysis was conducted using a Thermoscientific Nicolet 6700 spectrometer (Thermo Fisher Scientific Inc., Waltham, MA, USA) over a spectral range of 400–4000 cm^−1^. Thermogravimetry (TG) tests were performed in an N_2_ atmosphere using a Stanton STA 781 analyser (Stanton Redcroft Limited, London, UK), heating the samples to 1000 °C at a rate of 10 °C∙min^−1^.

Based on the TG data, the corresponding percentages of mass loss for Friedel’s salt (Ca_4_Al_2_(OH)_12_Cl_2_⋅4H_2_O), calcium monocarboaluminate (Ca_4_Al_2_(OH)_12_CO_3_⋅5H_2_O), portlandite (Ca(OH)_2_) and calcite (CaCO_3_) were calculated, taking into account the main temperature ranges in which each was detected. Equations (2)–(5) set out the calculation carried out:

(2) $$\%\mathrm{Friedel}\;\mathrm{salt}=\frac{\left({\mathrm{mass}}_{260℃}-{\mathrm{mass}}_{320℃}\right)*\mathrm{molecular}\;{\mathrm{mass}}_{\mathrm{Friedel}\;\mathrm{salt}}}{4*\mathrm{molecular}\;{\mathrm{mass}}_{H_{2}O}}$$

(3) $$\%\mathrm{Calcium}\;\mathrm{monocarboaluminate}\;(\mathrm{Mc})=\frac{\left({\mathrm{mass}}_{320℃}-{\mathrm{mass}}_{380℃}\right)*\mathrm{molecular}\;{\mathrm{mass}}_{\mathrm{Mc}}}{5*\mathrm{molecular}\;{\mathrm{mass}}_{H_{2}O}}$$

(4) $$\%\mathrm{Portlandite}=\frac{\left({\mathrm{mass}}_{400℃}-{\mathrm{mass}}_{500℃}\right)*\mathrm{molecular}\;{\mathrm{mass}}_{\mathrm{Ca}{\left(\mathrm{OH}\right)}_{2}}}{\mathrm{molecular}\;{\mathrm{mass}}_{H_{2}O}}$$

(5) $$\%\mathrm{Calcite}=\frac{\left({\mathrm{mass}}_{600℃}-{\mathrm{mass}}_{800℃}\right)*\mathrm{molecular}\;{\mathrm{mass}}_{{\mathrm{CaCO}}_{3}}}{\mathrm{molecular}\;{\mathrm{mass}}_{CO_{2}}}$$

XRD was carried out on a D8Advance diffractometer of Bruker (Bruker AXS GmbH, Karlsruhe, Germany) under the following measurement conditions: 40 kV generator voltage, 30 mA generator current, 6 mm variable divergence slit, 2 s time per step, 0.01981° step size and a 2θ range of 5–60°. Chloride content at different depths was chemically quantified using a portable XRF device (Olympus X-Innov-X 2000, Woburn, MA, USA) with an excitation source of 4W Au or Ta anode X-ray tube and a detector Si-PIN Diode.

## 3. Results and Discussion

### 3.1. Chloride Profiles

**Figure 2:**
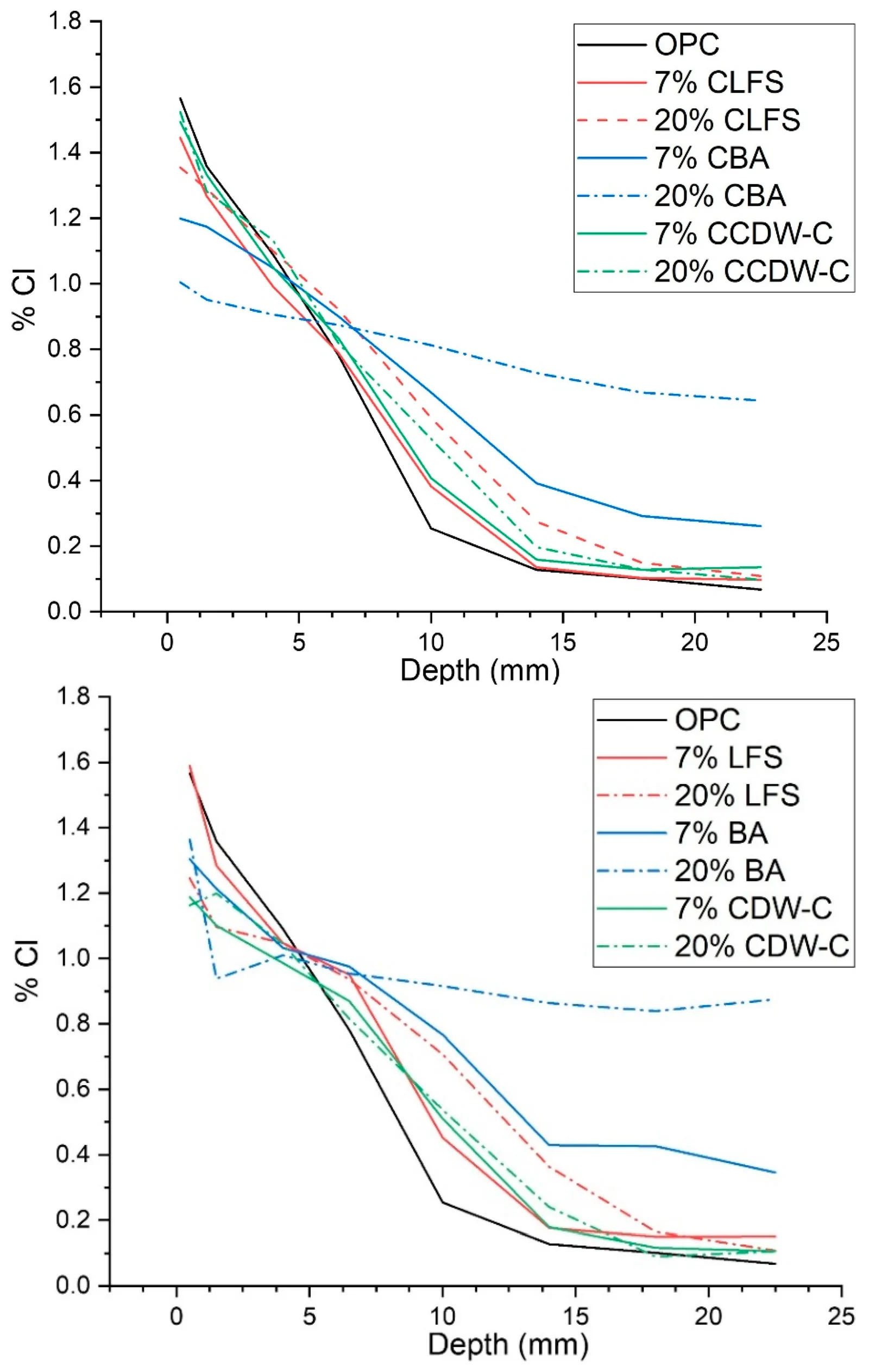
Chloride concentration profiles versus exposure depth: (**top**) carbonated and (**bottom**) non-carbonated waste materials alongside the OPC reference mortar.

Figure 2 presents the average chloride profiles of 13 mortar formulations containing either 7% or 20% carbonated (top) and 7% or 20% non-carbonated (bottom) waste materials. In all cases, chloride concentration declines progressively with depth, ranging from 1.6–1.0% at the exposed surface (0.5 mm) to as low as 0.2% in the interior (22.5 mm). The OPC reference matrix exhibits a sharp drop between 8 mm and 10 mm, characteristic of moderate penetration depth. The eco-mortars alter this behaviour depending on the waste type and replacement percentage.

In general, increasing the replacement percentage from 7% to 20% in carbonated waste materials (Figure 2 (top)) does not consistently enhance resistance to chloride penetration. While the 20% CLFS and 20% CCDW-C mortars show comparable or slightly improved performance relative to OPC, this improvement is not markedly superior in all the depths. In contrast, the 20% CBA mortars exhibit the poorest behaviour, maintaining relative high chloride concentrations close to 0.7%, which indicates higher permeability and deeper ion diffusion. A similar trend is observed in non-carbonated waste materials: the mortars incorporating LFS and CDW-C generally improve resistance to chloride diffusion, whereas those with BA replacement remain more vulnerable to chloride penetration. This behaviour may be related to differences in the microstructure and transport properties of the BA-based blends, which facilitate chloride ingress compared with the other waste-containing mortars.

The chloride profiles reveal that prior carbonation generally reduced chloride diffusion in the eco-blended mortars relative to their non-carbonated counterparts. These observations are consistent with a beneficial effect of CO_2_-induced mineralisation on chloride transport resistance, although the underlying mechanisms require further investigation through direct characterisation of pore structure, pore solution chemistry and chloride-binding behaviour.

Table 3 lists the chloride percentages determined by XRF (Section 2.3.3) at the two exposures depths of 0.5 mm and 22.5 mm after 90 days of chloride exposition (C_0.5,90_ and C_22.5,90_). It also shows the calculated chloride content at the exposed mortar surface (C_0_) and the non-steady-state diffusion coefficient D (cm^2^∙s^−1^) for each mortar under study using Fick’s second law (Equation (1), Section 2.3.3).

The values in Table 3 show that the OPC mortar has a diffusion coefficient of 4.350 × 10^−8^ cm^2^∙s^−1^ and a surface chloride concentration (C_0_) of 1.646%, thus falling within the expected range for conventional mortars and providing a suitable benchmark. The mortars containing 7% LFS, CLFS, CDW-C and CCDW-C exhibit diffusion coefficients of the same order of magnitude as the OPC (10^−8^ cm^2^∙s^−1^) and slightly lower C_0_ values, indicating that these waste materials do not increase chloride penetration susceptibility and, in several cases, even reduce it. The high R^2^ values obtained for these blends (≈0.97–0.99) confirm a good fit with the diffusion model, suggesting predominantly diffusive chloride transport and a relatively homogeneous microstructure.

In the case of the CDW-C and CCDW-C mortars, the D values range from 5.8 × 10^−8^ to 8.9 × 10^−8^ cm^2^∙s^−1^ respectively and have C_0_ values of around 1.27–1.55% by weight of cement (Table 3). The relative narrow range of diffusion coefficients suggests that the incorporation of concrete-based waste has a limited effect on chloride transport compared with other recycled additions. Although all mixtures exhibited higher D values than OPC, carbonation treatment consistently reduced the diffusion coefficients of the CCDW-C mortars. This behaviour suggests that prior carbonation contributes to microstructural refinement, thereby limiting chloride ingress by reducing pore connectivity and accessible pore volume.

In contrast, the biomass ash (BA and CBA) blends behave markedly differently. While the samples with 7% replacement content show diffusion coefficients moderately above those of OPC (1.6–2.0 × 10^−7^ cm^2^∙s^−1^ in contrast to 4.3 × 10^−8^), the 20% replacements exhibit a sharper increase, reaching 1.35 × 10^−6^ cm^2^∙s^−1^ for the CBA blend and up to 2.34 × 10^−6^ cm^2^∙s^−1^ for the BA blend. These coefficients, which are up to two orders of magnitude greater than those of OPC and the other waste materials, indicate a substantially higher susceptibility to chloride ingress. The poorer chloride transport performance observed in the BA- and CBA-based mortars is likely influenced by several interacting factors, including the higher replacement level, the characteristics of the biomass ash and changes in the hydration products. In particular the high SO_3_ content of the 20% CBA mortar (4.82%) compared to the reference OPC (3.14%) and the 20% CLFS and CCDW-C mortars (2.91% and 2.69%, respectively) [48] may influence the phase assemblage and the stability of sulphate- and chloride-bearing hydrates. This SO_3_ content tends to form a complex with the cement’s C_3_A to produce ettringite, thereby reducing Friedel’s salt formation [52]. However, the relative contribution of these mechanisms could not be determined independently in the present study.

Furthermore, the low coefficient of determination obtained for the 20% BA mortar (R^2^ = 0.610) indicates a poor fit of the experimental chloride profile to Fick’s diffusion model. This behaviour suggests that chloride transport in this formulation may be affected by factors not fully accounted for by the assumptions of homogeneous Fickian diffusion, such as material heterogeneity and complex transport processes, thereby reducing the reliability of the calculated diffusion coefficient.

From a chemical perspective, chloride ingress is governed not only by diffusion through the pore network but also by interactions between chloride ions and hydrated cement phases. In particular, chloride ions may react with monosulphoaluminate (AFm type) phases, including monocarboaluminate, forming chloride-bearing AFm phases such as Friedel’s salt. These reactions reduce the concentration of free chlorides in the pore solution and contribute to chloride immobilisation within the cementitious matrix. Consequently, the measured chloride profiles reflect the combined effects of chloride transport and chemical binding processes.

Overall, the results show that LFS-, CLFS-, CDW-C- and CCDW-C-containing mortars exhibit a considerably more favourable chloride transport behaviour that the biomass ash mixtures. Although their apparent chloride diffusion coefficients were generally higher than that of OPC, they remained within a relatively narrow range and showed a stable response with increasing replacement level. Furthermore, prior carbonation consistently reduced the diffusion coefficients of both the LFS and CDW-C mortars, highlighting its beneficial effect on chloride ingress resistance. Conversely, biomass ash, particularly at high replacement levels, significantly increased chloride diffusivity and resulted in a marked deterioration of durability-related performance.

These findings reveal that matrices with 20% waste material replacement content generally exhibited higher chloride diffusion coefficients than those with 7% replacement content, regardless of whether the waste materials had been carbonated. To better understand the microstructural factors governing this behaviour, the FTIR, XRD and DTG analyses were focused on the formulations with higher replacement percentages (20%), where the differences in chloride transport performance were most pronounced.

### 3.2. Mineralogical Analysis of the 20% Blended Mortars Using FTIR, DTG and XRD

To monitor the mineralogical changes induced by chloride attack in the non-steady-state ponding test and the reaction with the cement’s mineralogical phases, the techniques described in Section 2.4 were employed: Fourier transform infrared (FTIR) spectroscopy, X-ray diffraction (XRD) and thermogravimetry (TG/DTG). The analyses focused on the mortars with 20% carbonated and non-carbonated waste material replacement content at exposure depths of 0.5 mm and 22.5 mm.

#### 3.2.1. FTIR Analysis

**Figure 3:**
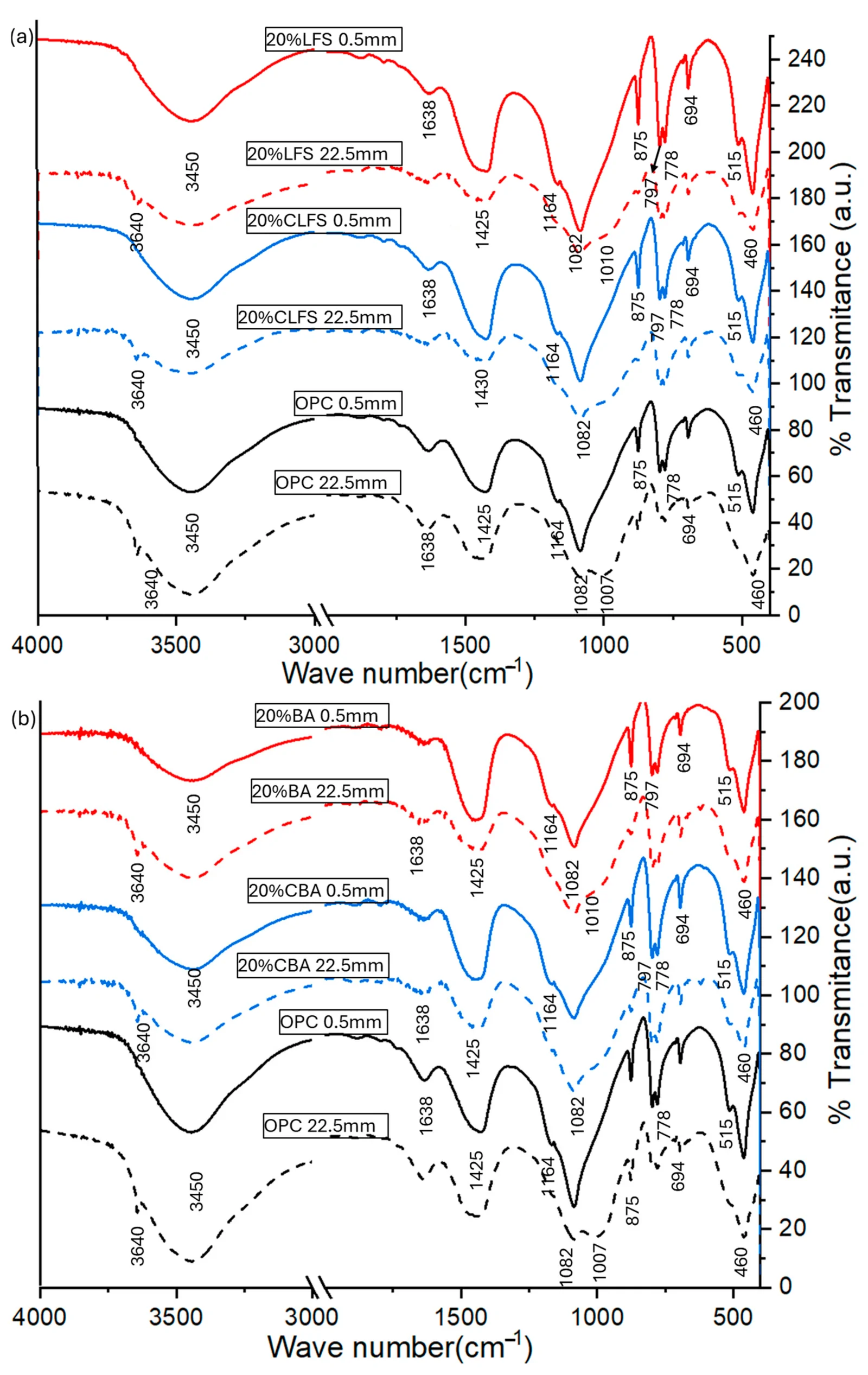

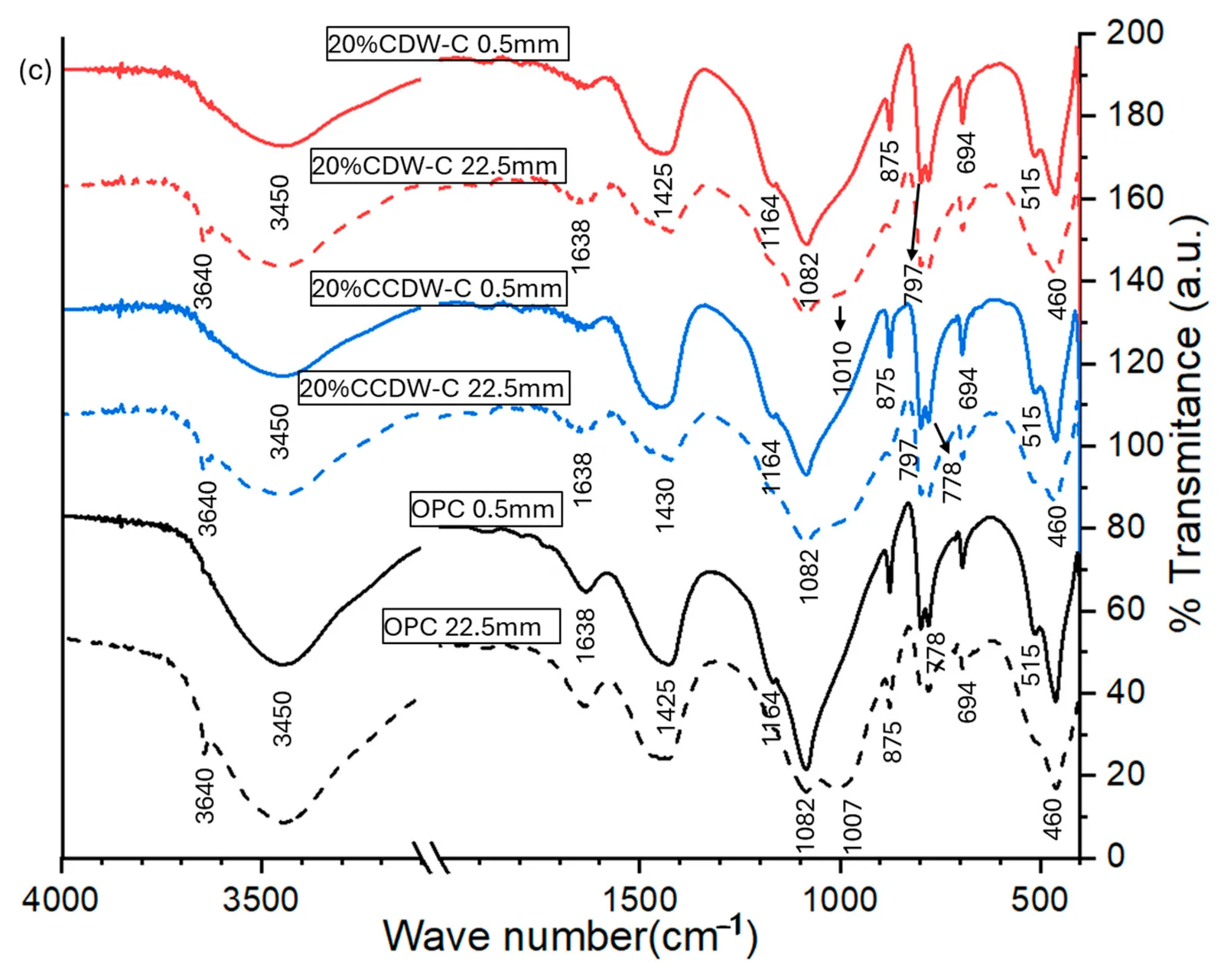
FTIR spectra of carbonated and non-carbonated mortars at exposure depths of 0.5 mm and 22.5 mm: (**a**) 20% LFS/CLFS; (**b**) 20% BA/CBA; and (**c**) 20% CDW-C/CCDW-C.

Figure 3 shows the FTIR spectra obtained for all the matrices under study: white ladle furnace slag (Figure 3a: CLFS and LFS), biomass ash (Figure 3b: CBA and BA) and construction and demolition waste (Figure 3c: CCDW-C and CDW-C). The spectra for the OPC reference mortar, which was also subjected to the same chloride attack, are included in all cases.

The 3700–3000 cm^−1^ region corresponds to O–H vibrations in hydroxyl groups and bound water. The OPC exhibits intense bands around 3640 cm^−1^, attributable to portlandite (Ca(OH)_2_) in the innermost layer (22.5 mm), and at 3450 cm^−1^, corresponding to hydration water at both depths. In contrast, the blended mortars show a marked decrease in the intensity of these signals, suggesting lower portlandite content consistent with pozzolanic reactivity in the waste materials—particularly the carbonated ones—that promotes Ca(OH)_2_ consumption during the formation of secondary hydration products.

The band at 1634 cm^−1^ assigned to H–O–H vibration in the water is sharply defined at a depth of 0.5 mm on the exposed surface of all the mortars, indicating greater retention of physically adsorbed water and differences in hydrated gel structure.

Characteristic bands associated with the formation of carbonates—calcite (CaCO_3_) and dolomite (CaMg(CO_3_)_2_)—and calcium monocarboaluminate (Ca_4_Al_2_(OH)_12_CO_3_·5H_2_O) in these materials appear in the 1400–1450 cm^−1^ (ʋ_3_ vibration) and 875–870 cm^−1^ (ʋ_4_ vibration) regions of the carbonate group. These signals are more intense in the 0.5 mm deep surface layers owing to the accumulation on the surface of carbonation products resulting from the interaction between Ca(OH)_2_ and atmospheric CO_2_.

The intense band at around 1082 cm^−1^ is mainly attributed to the Si–O stretching vibrations from silicate-containing phases, including C-S-H gels and quartz. It is accompanied by a shoulder at 1164 cm^−1^ assigned to the S–O bond of the sulphate group in phases such as ettringite (Ca_6_Al_2_(SO_4_)_3_(OH)_12_·26H_2_O), calcium monosulphoaluminate (Ca_4_Al_2_(SO_4_)(OH)_12_·6H_2_O) and gypsum (CaSO_4_·2H_2_O) at the 0.5 mm exposure depth.

The bands at 797 and 778 cm^−1^, together with those observed at 694, 515 and 460 cm^−1^, are associated with Si-O vibrational modes in crystalline quartz (SiO_2_). The doublet at 797 and 778 cm^−1^ is characteristic of quartz and is attributed to symmetric Si–O stretching vibrations (ν), while the lower-wavenumber bands correspond to Si-O bending (δ) modes. The greater intensity and sharper definition of these bands in the 0.5mm surface layer reflect the contribution of silica-rich phases derived from the standard sand and waste materials. In addition, the two lower-intensity bands are attributable to the vibrations of the Ca–O bond’s lattice.

The reduction in the intensity and broadening of the signals in the 1164–1082 cm^−1^ (S–O and Si–O bonds in the C-S-H gel) and 3640–3450 cm^−1^ regions (O–H bonds) in the blended mortars are attributed to the formation of secondary reaction products resulting from the waste materials’ increased pozzolanic activity.

The presence of chloride ions and the decrease in aqueous phase pH—processes accelerated by atmospheric CO_2_ ingress—acidify the system, altering the environment of the S–O and O–H bonds. These conditions could favour formation of Friedel’s salt through the synergy between kinetic chemical and thermodynamic processes in the mortars.

First, ettringite converts to calcium monosulphoaluminate. Second, chlorides negatively affect the stability of the portlandite (Ca(OH)_2_), hindering its formation. This effect is intensified by carbonation of the portlandite, which further reduces the system’s alkalinity and promotes the formation of phases capable of incorporating chlorides and carbonate groups.

The stability and transformation of these hydration products are governed by the interaction between hydrated sulfoaluminate phases, chloride ions, atmospheric CO_2_ and pore solution chemistry. The higher chloride concentration in the outer 0.5 mm layer may promote transformations within the AFm phase assemblage, favouring the development of chloride- and carbonate-bearing phases, such as Friedel’s salt (Ca_4_Al_2_(OH)_12_Cl_2_·4H_2_O) and calcium monocarboaluminate (Ca_4_Al_2_(OH)_12_CO_3_·5H_2_O). These processes may contribute to chloride binding within the cementitious matrix [53,54].

#### 3.2.2. TG/DTG Analysis

**Figure 4:**
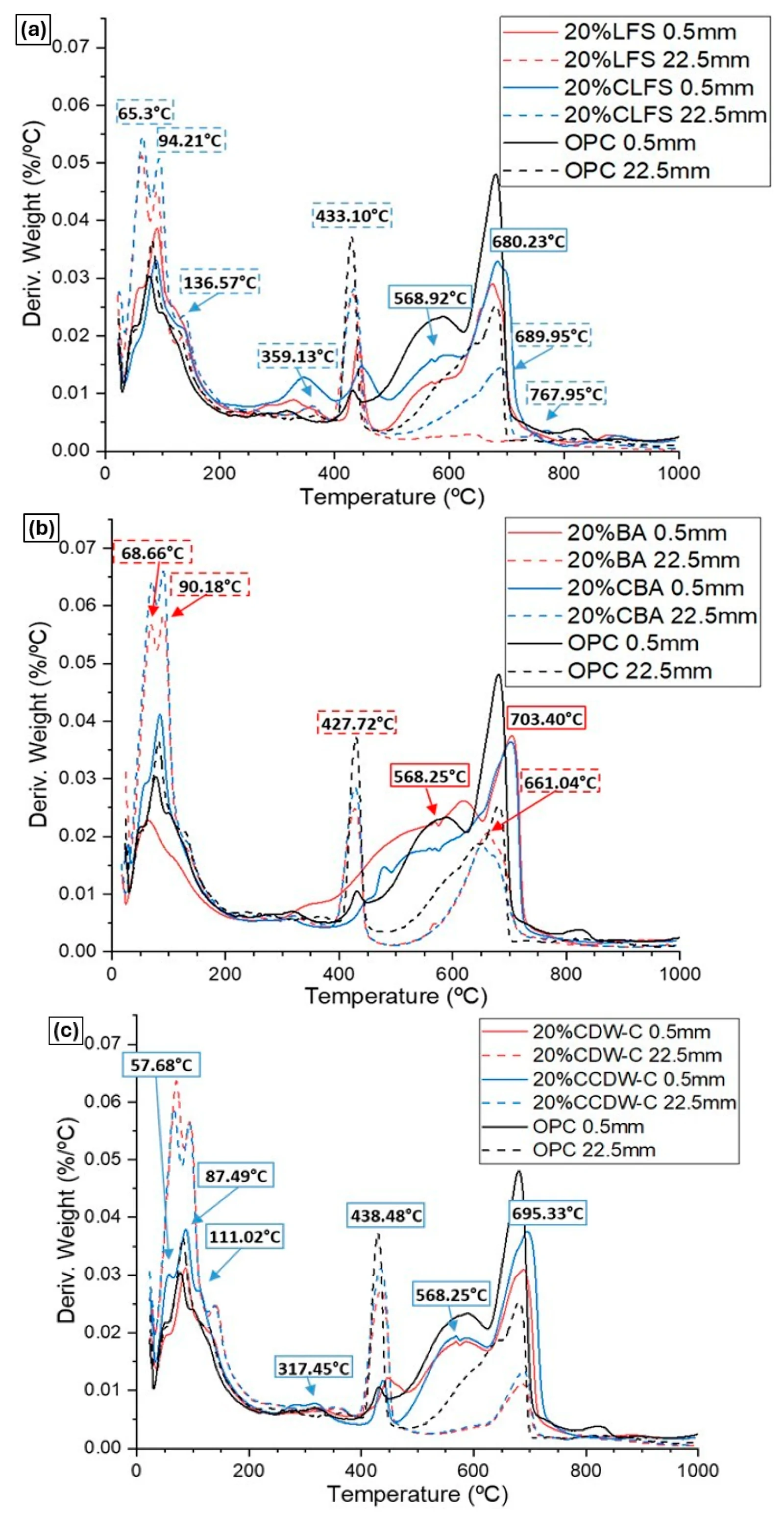
DTG curves for the mortars at exposure depths of 0.5 mm and 22.5 mm: (**a**) 20% LFS/CLFS; (**b**) 20% BA/CBA; and (**c**) 20% CDW-C/CCDW-C.

Figure 4a–c presents the first derivative of the mass loss curves (DTG) for selected 20% blended mortars made with carbonated and non-carbonated waste materials (LFS/CLFS, BA/CBA and CDW-C/CCDW-C) at exposure depths of 0.5 mm and 22.5 mm.

The DTG curves plot the rate of mass loss against temperature. In all the mortars, the rate of mass loss exhibits pronounced variations that reveal the presence of distinct chemical transformations. These variations correspond to the different decomposition stages that occur as temperature rises. The following detailed analysis of the transformations in each temperature range identifies the decomposition stages involved. This thermal segment study clearly distinguishes the kinetic mechanisms at play and sheds light on their contribution to the mortars’ overall behaviour.

**Figure 5:**
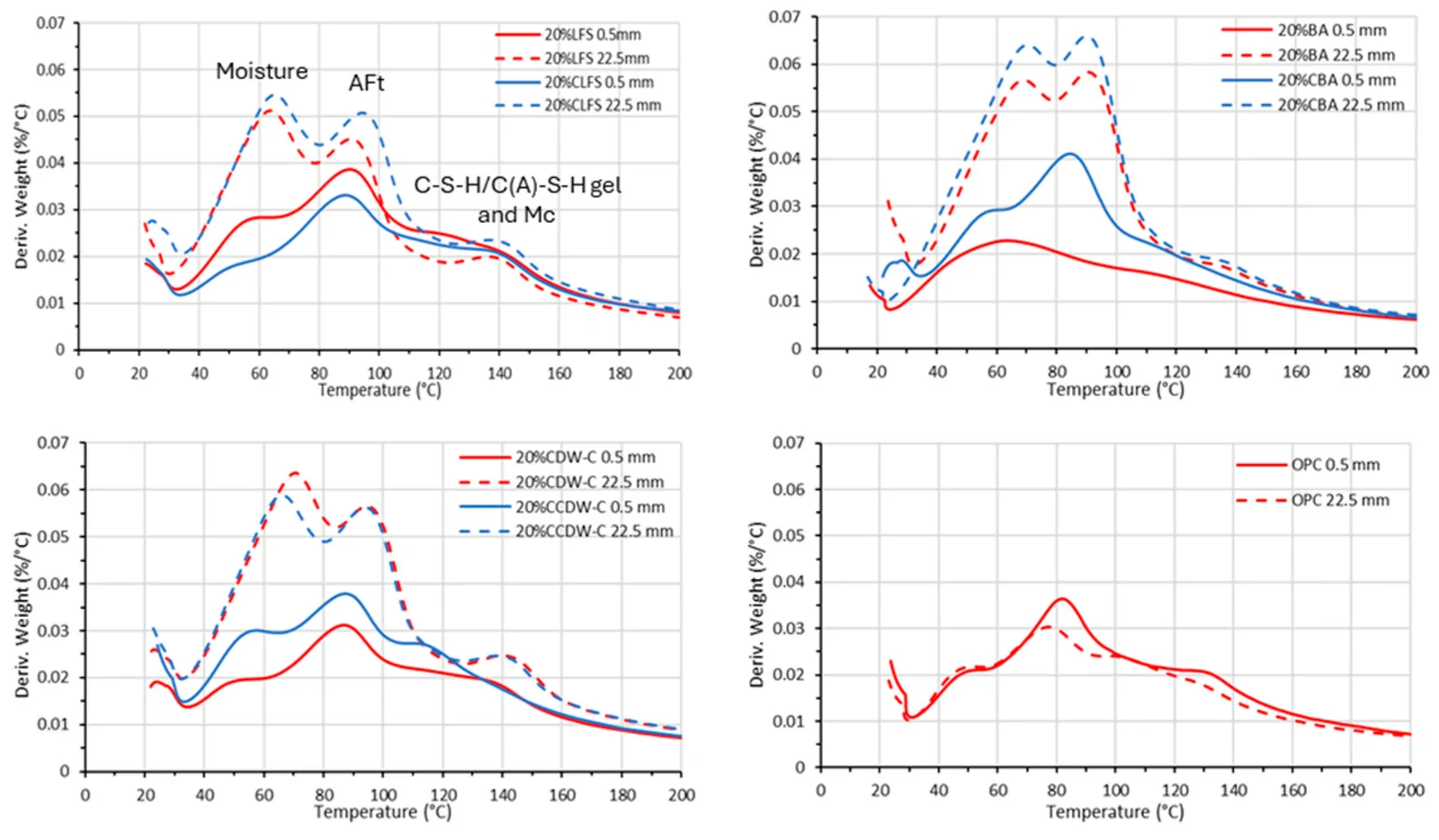
Increases in the DTG curves in the 0–200 °C temperature range.

In the 35–200 °C range (Figure 5), a broad signal with two peaks appears between 50 °C and 70 °C and between 80 °C and 110 °C. It is accompanied by a weaker signal between 110 °C and 160 °C (Figure 5). The first peak primarily reflects the free or adsorbed water in the samples. In all the blended mortars, this signal is most intense in the 22.5 mm exposure layer, indicating a higher degree of reaction in these mortars owing to the waste materials’ pozzolanic activity—particularly when carbonated (20% CLFS and 20% CBA). At higher temperatures (80–110 °C), another signal emerges, corresponding to the dehydration of hydration products such as ettringite. The only exception is the 20% BA mortar at the 0.5 mm exposure depth. The third interval (110–160 °C) corresponds to the dehydroxylation of C-S-H and C-(A)-S-H gels and the loss of five interlayer water molecules from the calcium monocarboaluminate (Mc) (Ca_4_Al_2_(OH)_12_CO_3_·5H_2_O), predominantly in the mortars with an exposure depth of 22.5 mm. Once again, the 20% BA and CBA mortars display differences in the presence and intensity of these bands. It is worth noting that at an exposure depth of 0.5 mm the mortars containing carbonated waste materials exhibit less intense signals in this range, particularly the 20% CCDW-C, which shows a higher degree of reaction owing to the presence of chloride ions, atmospheric CO_2_ and the pH decrease mechanism discussed in Section 3.2.1 [46,48].

**Figure 6:**
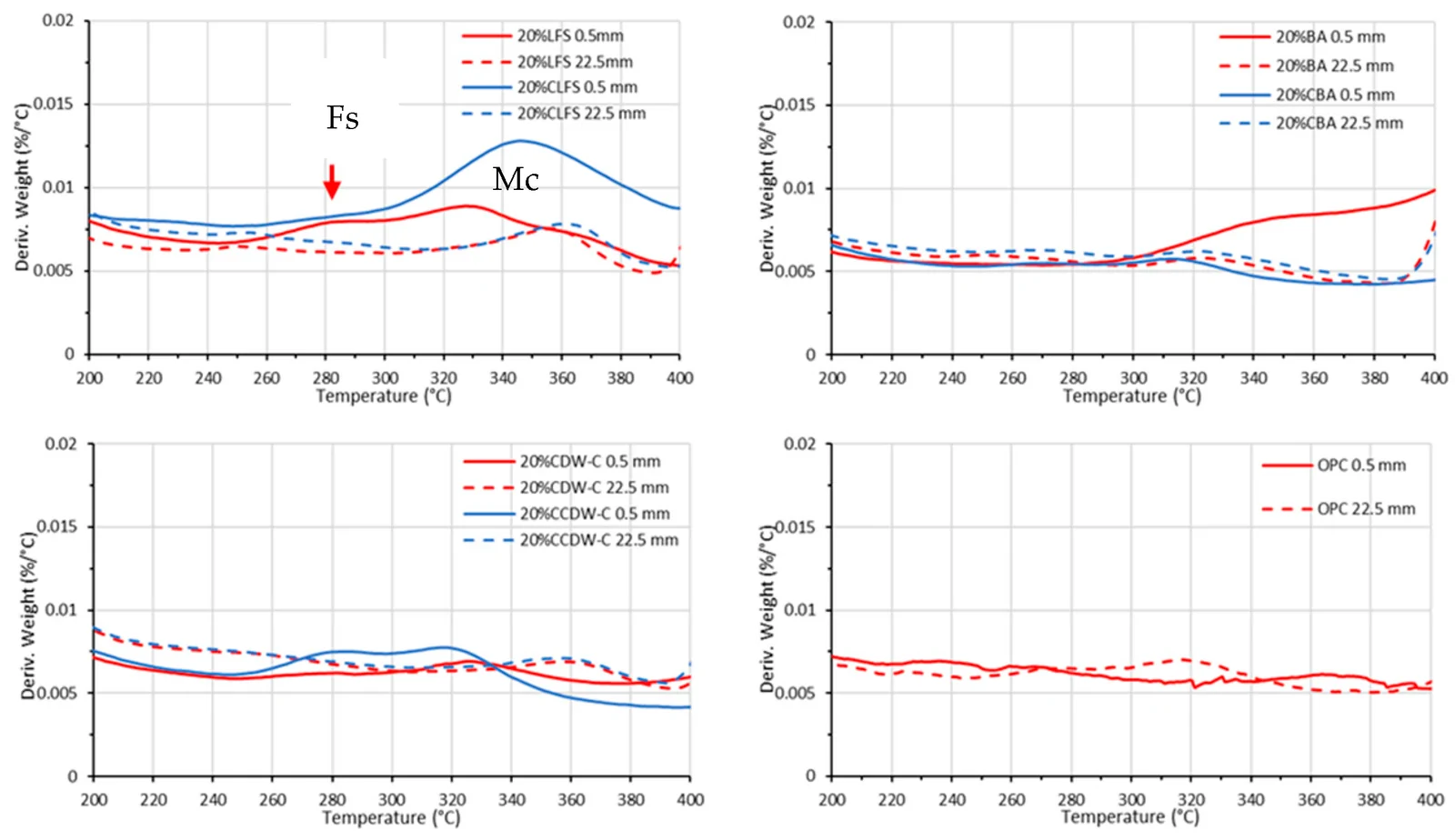
Increases in the DTG curves in the 200–400 °C temperature range.

In the 200–400 °C temperature range (Figure 6), the reaction rate remains practically constant (indicating no significant chemical transformations) except for two shoulders at 260–320 °C and 320–380 °C. The first corresponds to dehydroxylation of the water in the Friedel’s salt (Ca_4_Al_2_(OH)_12_Cl_2_⋅4H_2_O) in the 20% LFS and 20% CCDW-C mortars exposed to chlorides to a depth of 0.5 mm. The 320–380 °C range is associated with the loss of the five octahedrally coordinated water molecules in the calcium monocarboaluminate (Figure 6), primarily in the 20% CLFS mortar at a depth of 0.5 mm and in the 20% LFS, CLFS, CDW-C and CCDW-C mortars [46,48]. In the 20% BA and CBA mortars, the monocarboaluminate transformation band is faintly discernible at the 0.5 mm exposure depth.

**Figure 7:**
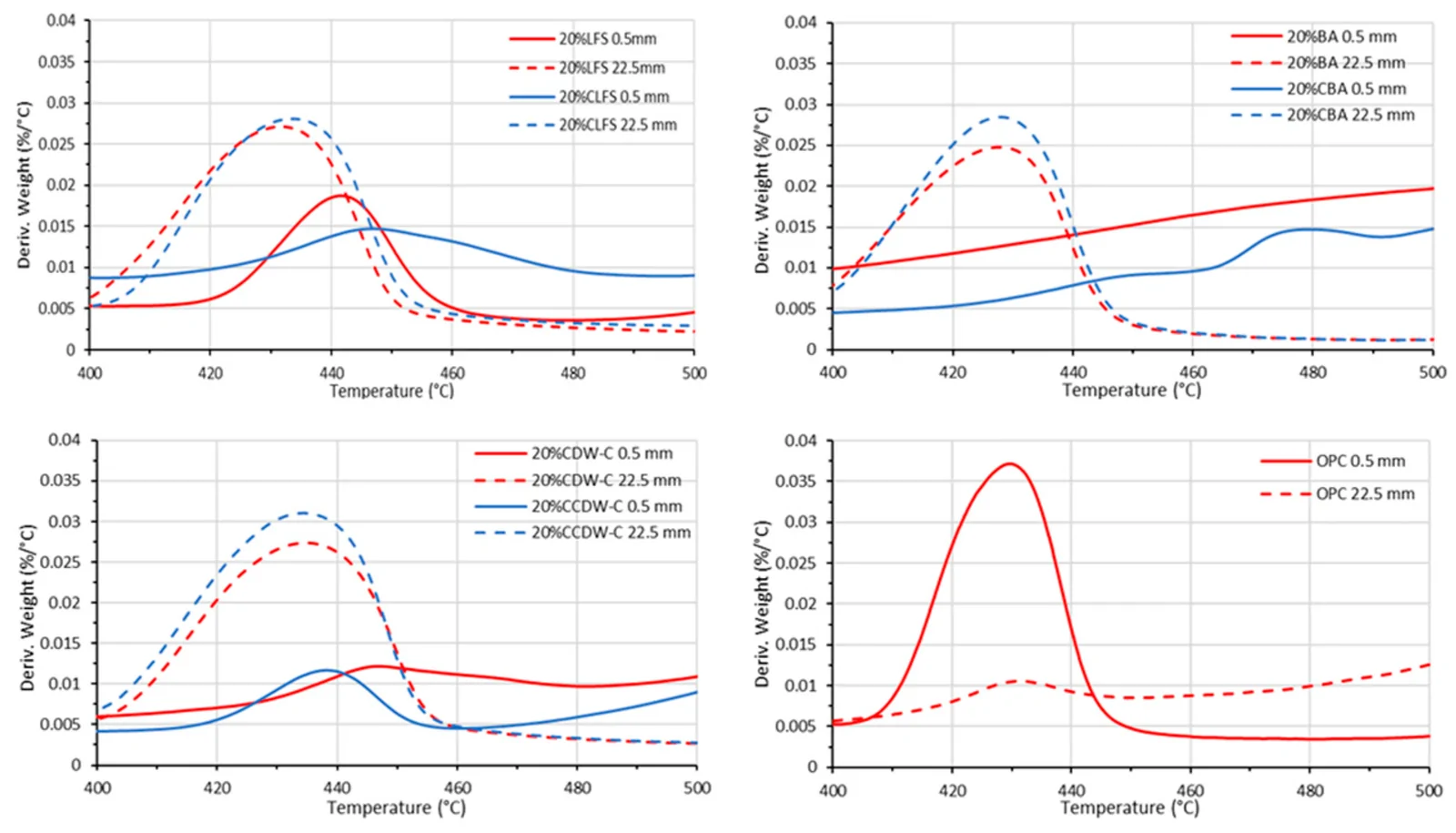
Increases in the DTG curves in the 400–500 °C temperature range.

In the 400–500 °C range, the chemical transformations are due to dehydroxylation of the portlandite (Figure 7). The formation rate of portlandite observed is higher in the blended mortars at the 22.5 mm exposure depth than in the OPC mortar at 0.5 mm. This behaviour stems from the pozzolanic nature of the waste materials, which promotes portlandite formation. In the 20% BA- and CBA-blended mortars at the 0.5 mm exposure depth, the reaction rate is nearly linear, indicating the absence of portlandite. The presence of chlorides and atmospheric CO_2_ instead favours its carbonation, as detected in the 500–800 °C range and discussed below.

**Figure 8:**
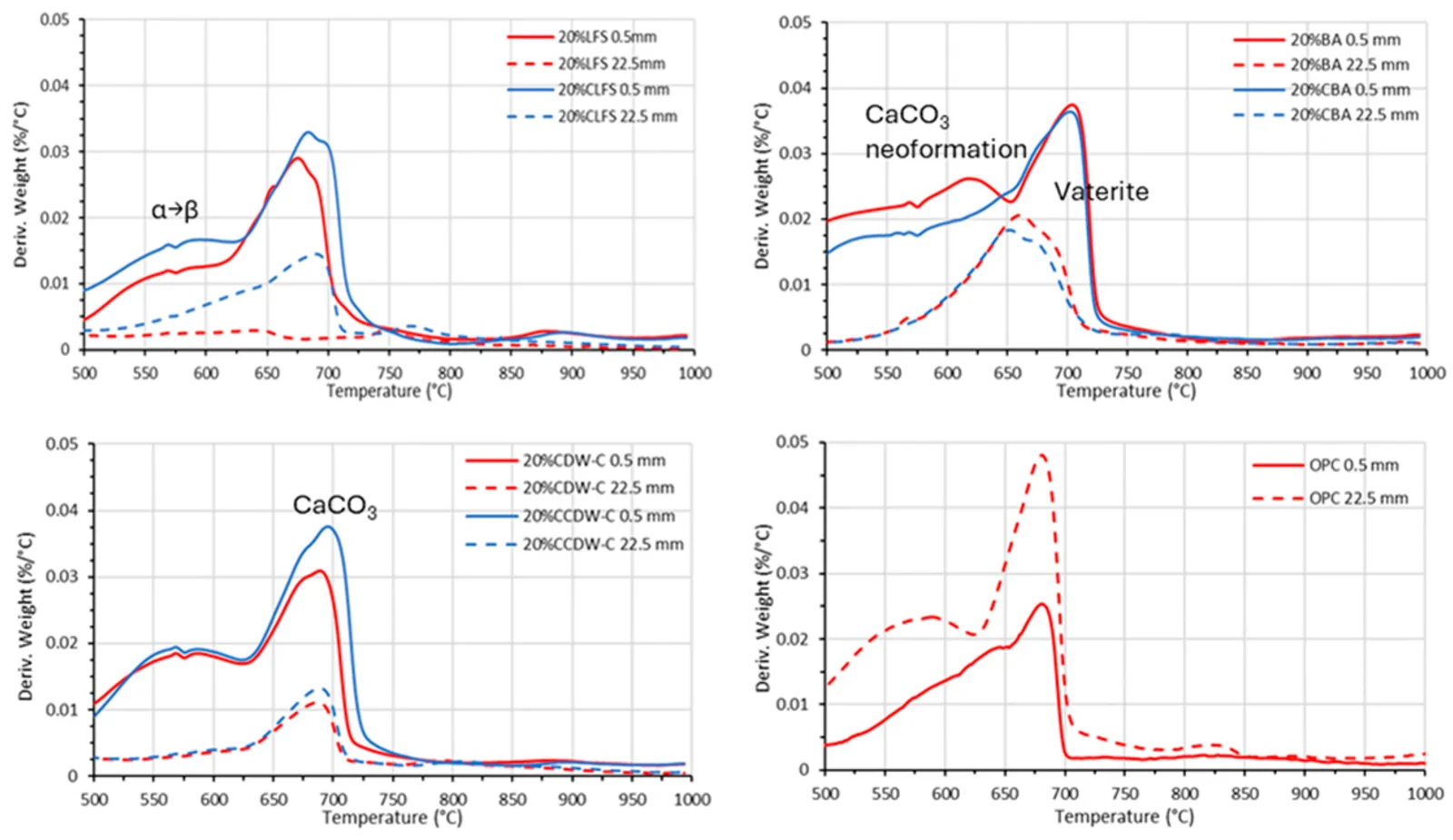
Increases in the DTG curves in the 500–1000 °C temperature range.

In the 500–800 °C range (Figure 8), the change in reaction rate reflects the chemical transformations associated with decarbonation processes in the mortars. Three well-defined peaks appear in this temperature interval, each attributable to distinct mineral phases. The 500–620 °C zone corresponds to the presence of slightly crystalline CaCO_3_ or newly formed calcium carbonate, along with calcium carboaluminate, as previously identified by FTIR (see Section 3.2.1). In the 650–675 °C region, at the 22.5 mm exposure depth the 20% BA and CBA mortars exhibit a peak linked to decarbonation of calcium carbonate in the form of vaterite and to recrystallisation of calcium chloroaluminate owing to interlayer water loss. Finally, in the 675–750 °C range, a high-intensity peak emerges, attributable to decarbonation of calcium carbonate in the form of calcite [55,56,57,58,59], although contributions from other carbonate-bearing phases cannot be excluded. It should be noted, however, that this temperature interval may also include contributions from other carbonate-bearing phases. Therefore, the results obtained in this region should be interpreted as indicative of carbonate decomposition rather than as an exclusive quantification of calcite. The latter transformation is particularly intense in the blended mortars at the 0.5 mm exposure depth, whereas in the OPC mortar it appears at the 22.5 mm depth. This difference stems from the higher reactivity of the mortars containing waste materials and is due to their pozzolanic activity, which promotes the formation and subsequent decarbonation of the phases involved.

The small band at 568 °C in the mortars with an exposure depth of 0.5 mm corresponds to α-quartz to β-quartz conversion.

Table 4 presents the weight losses recorded in the TG thermograms for the selected temperature intervals, along with the percentages of portlandite (400–500 °C), calcite (600–800 °C), monocarboaluminate (320–380 °C) and Friedel’s salt (260–320 °C) in Table 5.

Portlandite content rises with increasing depth, whereas calcite content exhibits the opposite trend. As expected, the highest calcite contents correspond to the mortars containing previously carbonated waste materials and to the newly formed calcite resulting from chloride attack. This observation aligns with the changes in the DTG curves.

#### 3.2.3. XRD Analysis

**Figure 9:**
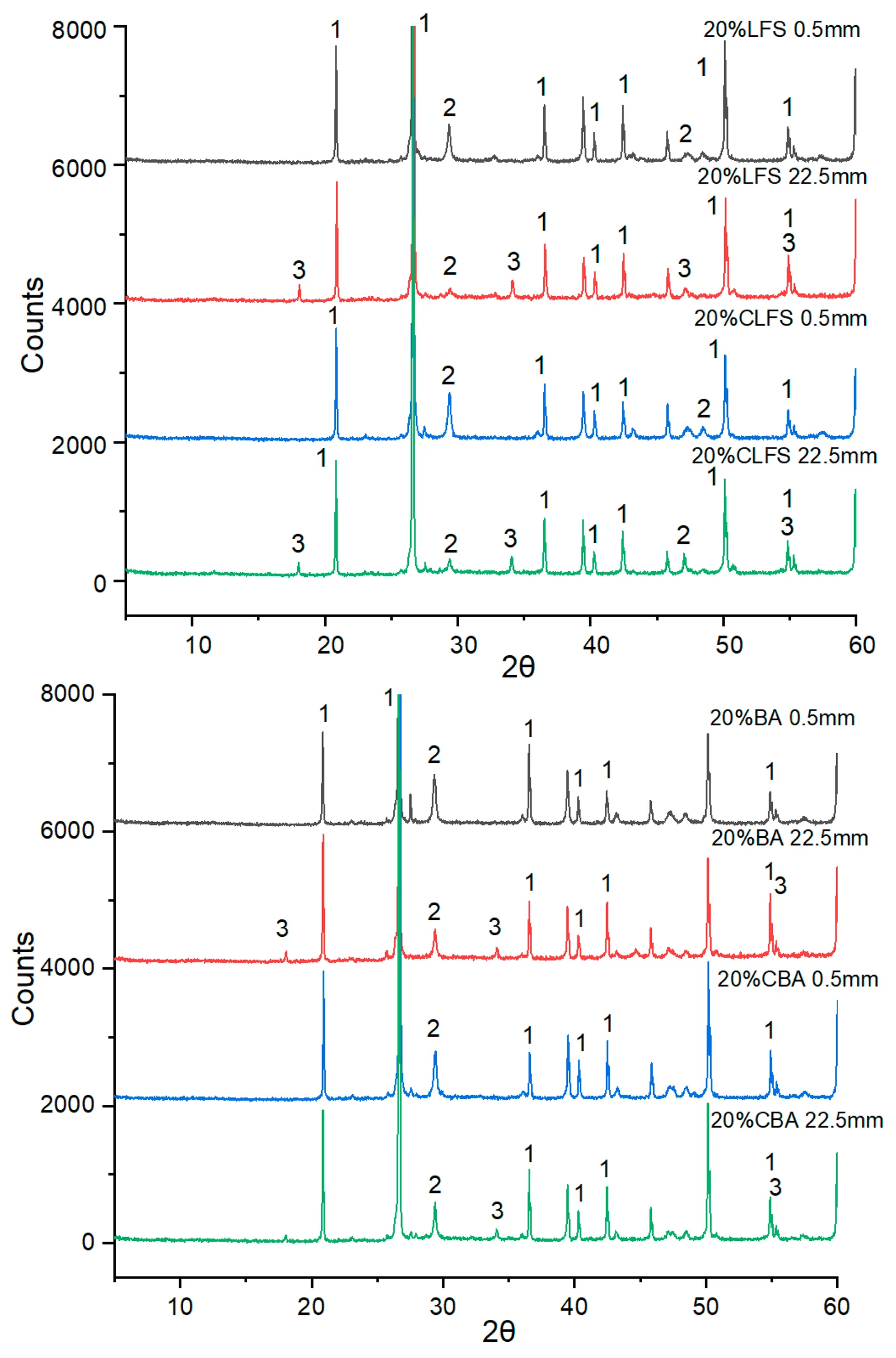

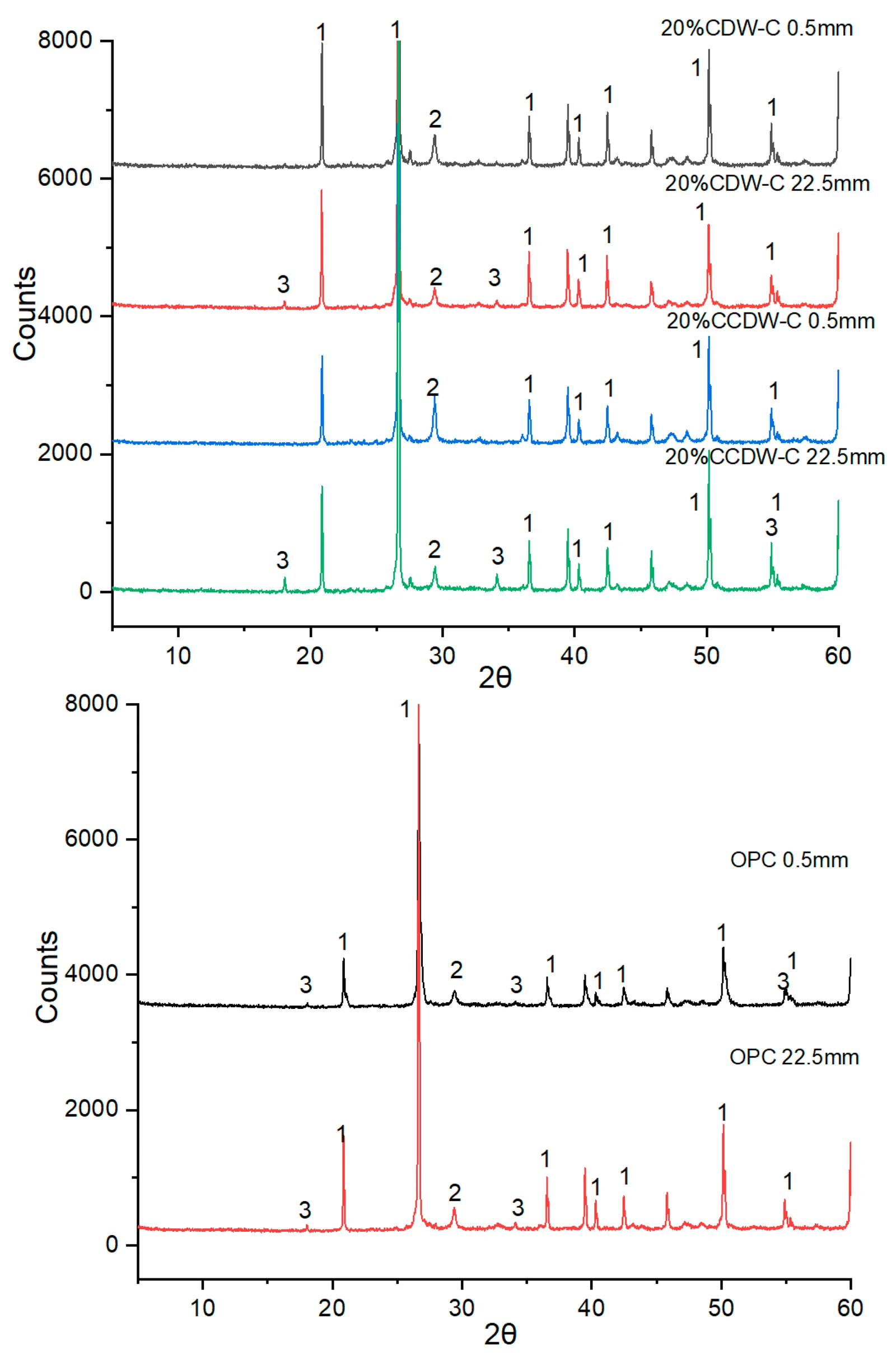
X-ray diffraction patterns of 20% blended mortars and OPC at the 0.5 mm and 22.5 mm exposure depths: 1—quartz (SiO_2_); 2—calcite (CaCO_3_); and 3—portlandite (Ca(OH)_2_).

**Figure 10:**
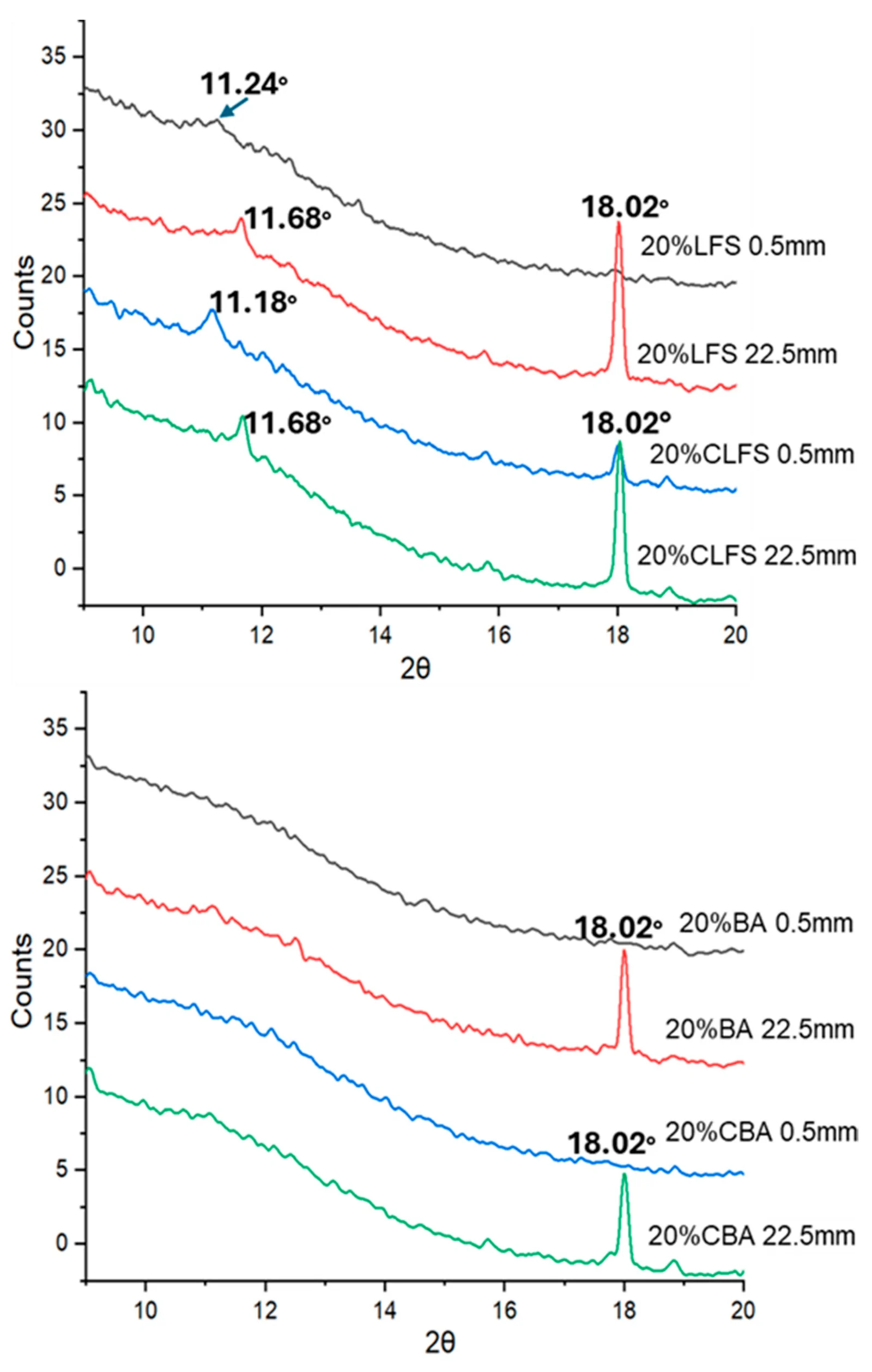

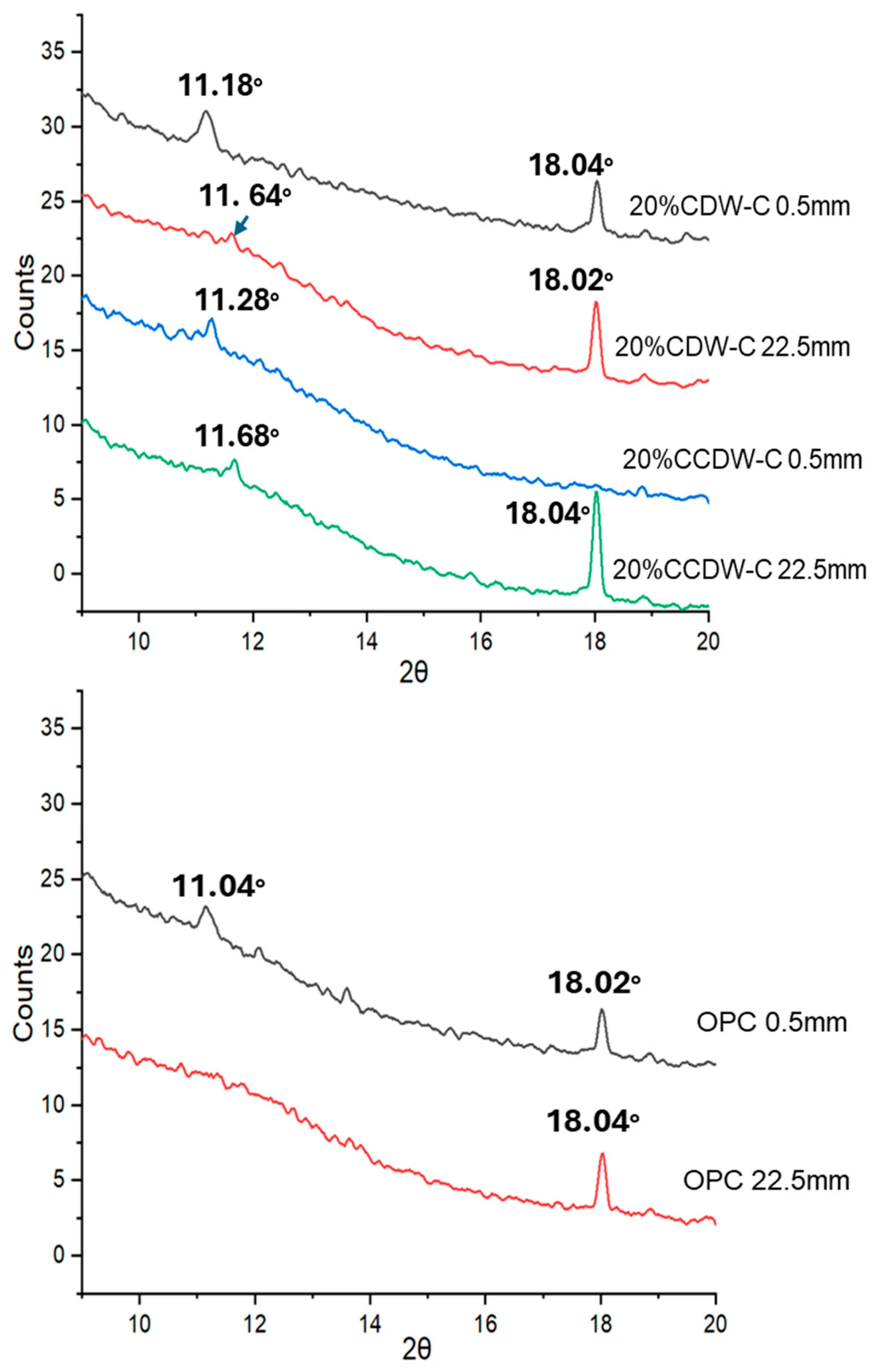
X-ray diffraction patterns of OPC and mortars containing 20% waste replacement after chloride exposure. Details of the 5-20° 2θ range showing the characteristic reflections used for the identification of Friedel’s salt (Fs) (11.04–11.28°), monocarboaluminate (Mc) (11.64–11.68°), and portlandite (CH) (18.02° and 18.04°).

To assess the mineralogical evolution of the materials after chloride exposure, Figure 9 presents the XRD diffractograms of the 20% blended mortars over the 5–60° (2θ) range at the 0.5 mm and 22.5 mm exposure depths. In addition, a magnified view between the 5° and 20° (2θ) region is included because the main and non-overlapping reflections of the AFm phases of interest, particularly Friedel’s salt and calcium monocarboaluminate, are concentrated within this interval (Figure 10). In all cases, a diffuse background is observed in this last range (2θ), reflecting the significant amorphous content of the binders. This feature is associated with the presence of poorly crystalline hydration products, mainly C-S-H gel phases, together with the vitreous fraction of the waste-derived precursors.

The full-range XRD diffractograms (5-60° 2θ) are dominated by quartz, calcite and portlandite reflections. Quartz, mainly associated with the siliceous aggregate, represents the most intense crystalline phase and largely governs the diffraction response of the mortars. Consequently, the identification of minor crystalline reaction products is challenging due to peak overlap and masking effects associated with the aggregate contribution. Therefore, the full-range diffractograms are primarily used to identify the major mineralogical constituents, whereas the evolution of AFm phases is assessed using the expanded 5–20° 2θ region in Figure 10, where the characteristic reflections of Friedel’s salt and monocarboaluminate are located.

The diffractograms show the characteristic reflection of portlandite (Ca(OH)_2_), with the peak located between 18.02° and 18.04° (2θ). It is attributable to the formation of a typical cement hydration product. The intensity of the peak declines markedly in the attacked surface layer (0.5 mm). In certain cases, namely 20% LFS (non-carbonated), 20% BA and CBA (both non-carbonated and carbonated) and 20% CCDW-C (carbonated), no clearly distinguishable crystalline portlandite reflections were observed. Around 11–12° (2θ), a set of low-intensity peaks appears in all the formulations. These signals are associated with the possible formation of AFm-type phases such as calcium monocarboaluminates and Friedel’s salt. According to the literature, the reflection located at approximately 11.04° (2θ) can be attributed to Friedel’s salt, while the one at approximately 11.68° (2θ) is associated with the calcium monocarboaluminate (Mc) phase, [11,60,61].

It is important to underline that the above-mentioned peak associated with the Friedel’s salt is mainly identified in the samples at an exposure depth of 0.5 mm, where the chloride ingress is expected to be more significant. In contrast, the calcium monocarboaluminate reflection is predominantly observed at an exposure depth of 22.5 mm in most formulations, with the exception of 20% BA, 20% CBA and the OPC reference mortar. This distribution is consistent with the phase assemblage expected under chloride exposure, where AFm phases may undergo transformations associated with chloride-binding processes. Similar observations have reported by previous authors [1], who indicated that monocarboluminate (Ca_4_Al_2_(OH)_12_CO_3_·5H_2_O) may progressively transform Friedel’s salt (Ca_4_Al_2_(OH)_12_Cl_2_·4H_2_O) through chloride incorporation. Consequently, the preferential identification of Friedel’s salt in the outer layer and the persistence of calcium monocarboaluminate at greater depths may be associated with a difference in chloride availability within the mortar specimens.

Overall, the XRD results provide qualitative evidence of the presence of the main crystalline phases and support the occurrence of AFm phase transformations associated with chloride exposure. However, owing to the complex multiphase nature of the mortars and the dominant contribution of quartz from the aggregate, the diffractograms are interpreted qualitatively and no quantitative phase assessment is derived solely from peak-intensity variations.

### 3.3. Durability Analysis

Based on the above data, durability analysis was conducted for a hypothetical structure built using these eco-materials and assuming the XS environmental exposure class defined in Eurocode 2 [19]. The analysis is intended solely as a comparative theoretical estimate of the relative performance of the different mortar formulations and should not be interpreted as an absolute service-life prediction. The calculations are based on the diffusion coefficients obtained from the chloride profiles after 90 days of exposure and assume simplified and constant exposure conditions. Consequently, the model does not explicitly account for the age dependence of chloride diffusivity, time-dependent surface chloride concentration, non-linear chloride binding, environmental variations, cracking, or coupled moisture transport.

**Figure 11:**
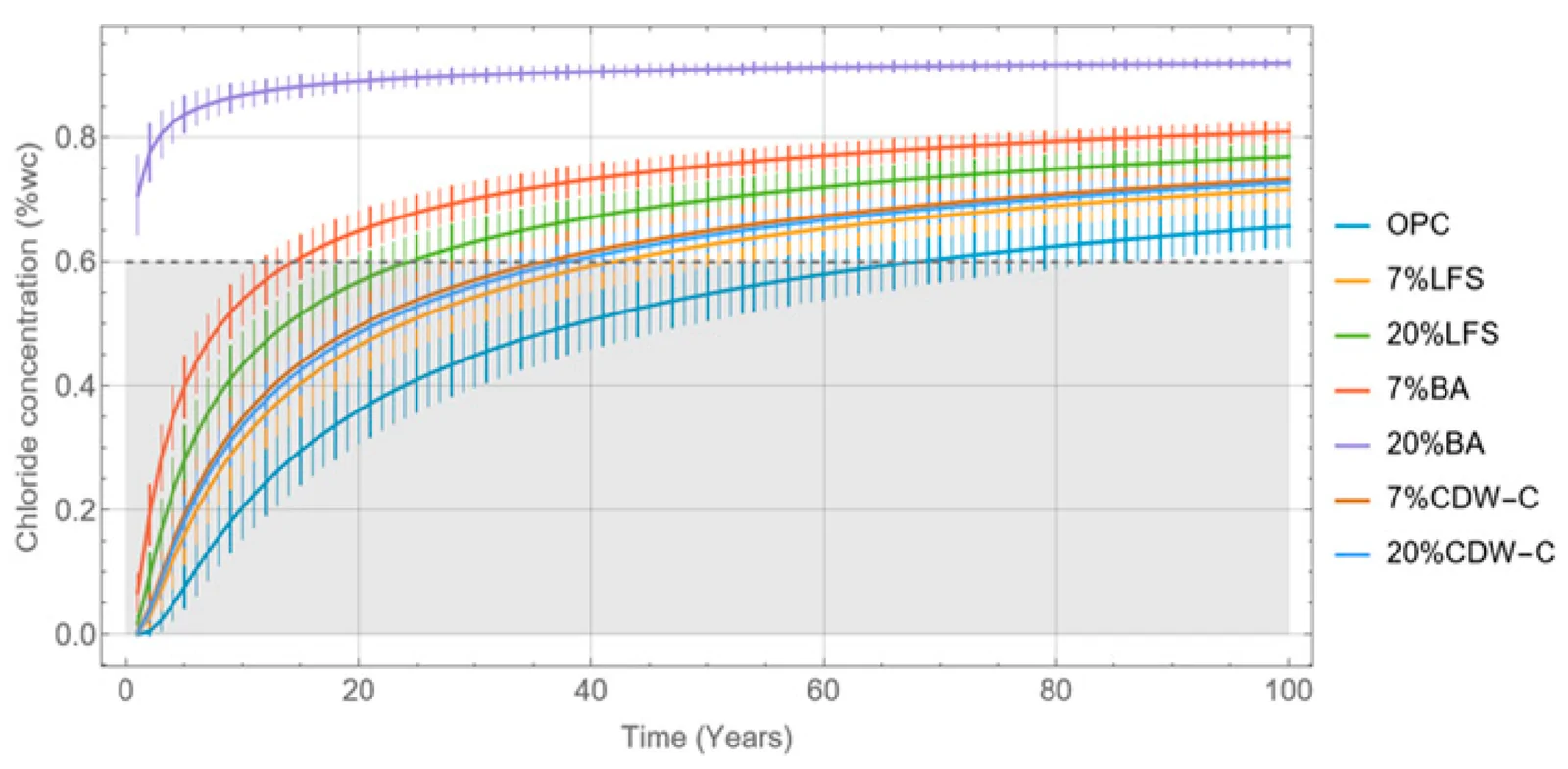
Changes over time in chloride concentration at steel reinforcement depth—non-carbonated samples.

**Figure 12:**
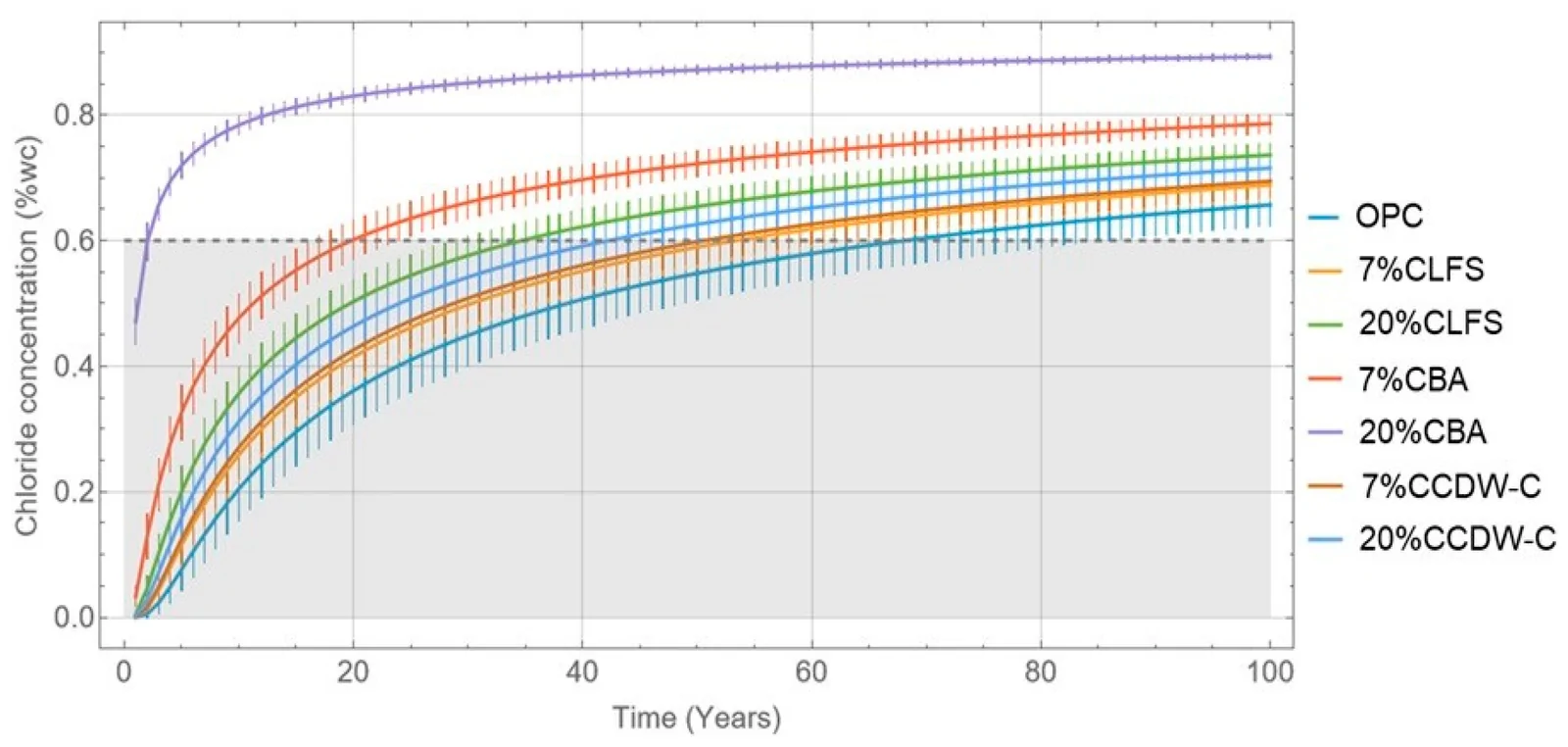
Changes over time in chloride concentration at steel reinforcement depth—carbonated samples.

**Figure 13:**
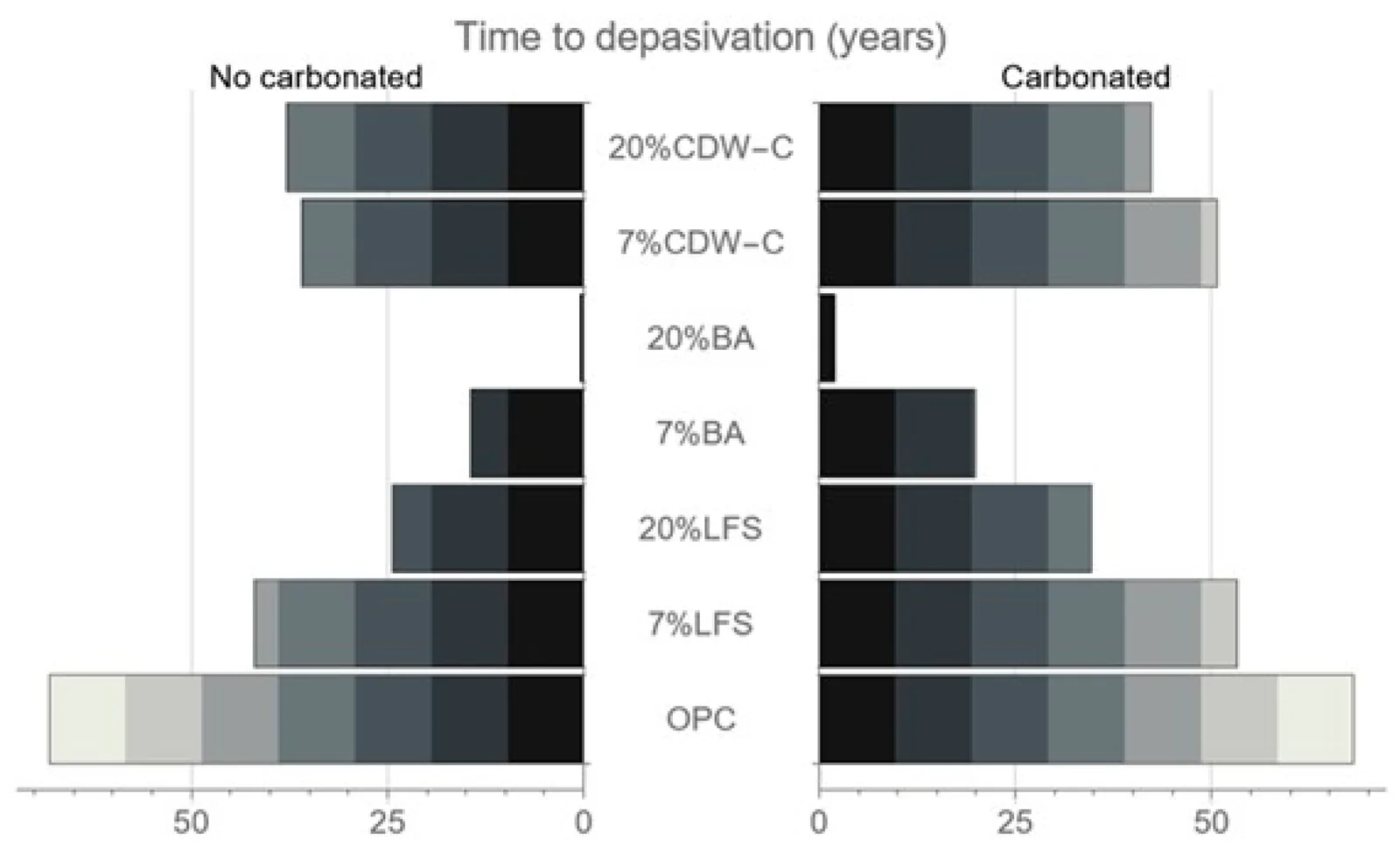
Time to depassivation.

For this scenario, the baseline parameters employed in the service-life calculations were as follows: surface chloride concentration of 0.95% wc and a nominal covering of the steel reinforcements of 65 mm, incorporating ±5 mm variability to account for construction tolerances. Using the error function solution as the solution to Fick’s second law referred to above (Equation (1)), the chloride concentration at the depth of the reinforcement was estimated [22]. To reflect the variability associated with both material properties and execution, the analysis considered (i) the variability in the diffusion coefficient derived from profile fitting and (ii) the variability in covering depth. The results, presented in Figure 11, Figure 12 and Figure 13 with error bars, show the changes in chloride concentration at the steel reinforcement depth for the blends containing non-carbonated waste materials in Figure 11. Chloride ions diffuse from the structure’s exterior toward the interior, and corrosion initiates when the chloride concentration at the reinforcement depth reaches 0.6% wc [19]. This threshold is marked in the figure since its intersection with the curves determines the moment of corrosion initiation. The behaviour varies widely, ranging from the extremely poor protective performance of the 20% BA mortar to the superior resistance exhibited by the OPC reference mortar. Figure 12 shows the results obtained for the mortars made with carbonated waste materials. In general terms, prior carbonation moderately extends the time to depassivation, indicating a slight improvement in chloride ingress resistance versus their non-carbonated counterparts. However, the replacement level exerts a more decisive influence: increasing the replacement content from 7% to 20% generally shortens the time to initiation. Figure 13 summarises the estimated time to depassivation, highlighting the critical case of the 20% BA/CBA, where the initiation time drops to just 1–2 years under the scenario considered.

Figure 13 shows the time to steel depassivation based on a critical chloride concentration of 0.6% wc and the changes in chloride concentration over time shown in the preceding figures. This figure distinguishes between the non-carbonated and carbonated samples, showing that carbonation has a favourable effect by marginally extending the time to depassivation. The replacement percentage, however, has a greater impact, producing a more pronounced reduction in the initiation time as the replacement content rises from 7% to 20%. The extreme case is the BA blend, where the initiation time falls from 14–19 years to just 1–2 years, meaning corrosion would begin within that latter period.

The poorer performance of the blended mortars containing this type of biomass ash is due to the combined effects of two factors, as discussed in Section 3.1. First, the ash’s low SiO_2_ (2.32%) and Al_2_O_3_ (0.57%) contents primarily exert a cement dilution effect. Second, the high sulphate content of the ash (SO_3_ = 10.35%) raises the sulphate level of the cement matrix (SO_3_ = 4.82% at 20% replacement, versus 3.14% for the reference OPC), which neutralises chloride binding by C_3_A, leaving high concentrations of free chloride in the pore solution and significantly reducing the service life [52]. The specific contribution of phase transformations related to sulphate, including possible ettringite formation, cannot be established from the present dataset and requires further mineralogical and dimensional stability investigation. Therefore, further assessment of dimensional stability related to the sulphate, including expansion measurements and phase evolution during prolonged curing, would be required before considering these formulations with biomass ash for practical applications.

## 4. Conclusions

This study on the chloride resistance and hence durability of eco-mortars made with previously carbonated alkaline waste materials yields the following key findings.

Exposure to chloride ions at a depth of 0.5 mm resulted in a reduction portlandite content, which can be associated with the formation of carbonated phases and carboaluminates. In addition, chloride ingress modified the C-S-H gel structure, as reflected in the broadening and reduced intensity of the Si–O and O–H bands, particularly in the blended mortars.

1. The presence of chloride ions and the accompanying decrease in pore solution alkalinity promoted the transformation of sulphoaluminate phases in the blended cement mortars. This process led to the formation of secondary products, mainly Friedel’s salt and calcium monocarboaluminate, especially in the zones most affected by chloride exposure (0.5 mm depth).
2. Calcite exhibits the opposite distribution pattern, being predominantly concentrated in the surface layer as a consequence of both the prior carbonation treatment applied to the waste materials and the neoformation of calcite induced during chloride exposure. Mortars incorporating previously carbonated waste materials showed enhanced mineralogical reactivity under chloride exposure compared with their corresponding non-carbonated counterparts. This behaviour was reflected by higher Ca(OH)_2_ consumption and an increased formation of chloride-binding capacity associated with the carbonation treatment.
3. The depletion of portlandite in the surface layer, together with the detection of Friedel’s salt at a depth of 0.5 mm and of calcium monocarboaluminate at 22.5 mm, supports the occurrence of a depth-dependent ion-exchange mechanism between carbonate and chloride ions. These findings highlight the combined influence of kinetic and thermodynamic factors that govern AFm phase stability in chloride-rich environments.
4. Prior carbonation generally enhanced durability under an XS exposure scenario by delaying the estimated depassivation of reinforcing steel, although the extent of his benefit strongly depended on the waste type and replacement level. The LFS/CLFS and CDW-C/CCDW-C delivered the best durability performance among the alternative binders. However, despite these improvements, the blended mortars still underperformed compared to OPC, underscoring the challenge and opportunity of further optimising sustainable cementitious materials.

The findings demonstrate the feasibility of using CO_2_-mineralised industrial waste materials as eco-supplementary cementitious materials (eco-SCMs), providing a dual environmental benefit through waste valorisation and carbon capture. Pre-carbonation was found to improve chloride-related durability indicators in several formulations when compared with their non-carbonated counterparts, although the extent of this benefit depends on the nature of the waste material and does not necessarily imply superior performance to OPC. Consequently, these materials represent a promising route towards more circular and lower-carbon cementitious systems while maintaining acceptable durability under chloride exposure conditions.

## 5. Limitations and Future Perspectives

Although the present study provides valuable insights into the chloride penetration behaviour and service-life performance of eco-cement mortars incorporating previously carbonated alkaline waste materials, several limitations should be acknowledged. The investigation was conducted under controlled laboratory conditions and focused on chloride ingress as the primary durability indicator. Consequently, the influence of other degradation mechanisms, such as sulfate attack, freeze–thaw cycles, carbonation after service exposure, or combined deterioration processes, was not evaluated.

Furthermore, the mineralogical characterisation was limited to qualitative phase identification, while quantitative approaches such as Rietveld refinement were beyond the scope of the current work. Future studies should therefore incorporate advanced quantitative mineralogical and microstructural characterisation techniques to better establish the relationships between carbonation-induced phase transformations, chloride-binding capacity, transport properties and long-term durability performance. In addition, extending this approach to other alkaline industrial residues would contribute to assessing the broader applicability of CO_2_ mineralisation as a pre-treatment strategy for the production of sustainable cementitious materials.

## Figures and Tables

**Table 1:** Chemical composition of the major oxides of the non-carbonated starting waste materials.

Waste	SiO_2_	CaO	MgO	SO_3_	K_2_O	Al_2_O_3_	Minorities	LOI
**LFS**	17.68	48.81	11.66	1.8	0.04	4.98	4.39	7.37
**BA**	2.32	39.61	3.12	10.41	17.53	0.57	2.47	21.34
**CDW-C**	43.81	27.13	1.17	0.97	2.23	6.32	2.59	15.63

**Table 2:** Quantification of the mineralogical phases of the non-carbonated starting waste materials (nd: not detected).

Minerals (%)	Chemical Formula	LFS	BA	CDW-C
Periclase	MgO	8	10	nd
Katoite	Ca_3_Al_2_(OH)_12_	nd	10	nd
Calcite	CaCO_3_	nd	12	17
Dolomite	CaMg(CO_3_)_2_	nd	11	nd
Ca-olivine	Ca_2_SiO_4_	10	-	nd
Portlandite	Ca(OH)_2_	9	13	nd
Gibbsite	Al(OH)_3_	6	nd	nd
Hydrogarnet	C_3_AH_5_	7	nd	nd
K-sulphate	K_2_SO_4_	nd	13	nd
Mica	*K*(Fe^2+^/Mg)_2_(*Al*/Fe^3+^/Mg/Ti)([Si/*Al*/Fe]_2_*Si*_2_*O*_10_)(*OH*/F)_2_	nd	nd	8
Quartz	SiO_2_	nd	nd	22
Plagioclase	(Na,Ca)[(Si,Al)AlSi_2_]O_8_	nd	nd	9
K-feldspar	K(AlSi_3_O_6_)	nd	nd	6
Am material	-	60	31	38
R_B_	-	19.6	17.3	17.3
X^2^	-	9.4	9.2	6.9

**Table 3:** Chloride concentration values at a depth of 0.5 and 22.5 mm after 90 days (C_0.5,90_, C_22.5,90_) (%), calculated surface chloride concentration (C_0_) (%), diffusion coefficient D (cm^2^∙s^−1^) and R^2^.

Sample	C_0.5,90_ (%)	C_22.5,90_ (%)	C_0_ (%)	D (cm^2^∙s^−1^)	R^2^
OPC	1.57	0.07	1.646 ± 0.058	4.350 × 10^−8^ ± 5.565 × 10^−9^	0.988
7% LFS	1.59	0.15	1.573 ± 0.075	6.542 × 10^−8^ ± 1.127 × 10^−8^	0.974
20% LFS	1.24	0.11	1.301 ± 0.056	1.252 × 10^−7^ ± 2.019 × 10^−8^	0.971
7% BA	1.30	0.35	1.306 ± 0.045	2.014 × 10^−7^ ± 2.783 × 10^−8^	0.971
20% BA	1.36	0.88	1.109 ± 0.078	2.342 × 10^−6^ ± 1.904 × 10^−6^	0.610
7% CDW-C	1.19	0.11	1.278 ± 0.059	8.787 × 10^−8^ ± 1.479 × 10^−8^	0.975
20% CDW-C	1.16	0.11	1.309 ± 0.053	8.874 × 10^−8^ ± 1.302 × 10^−8^	0.979
7% CLFS	1.44	0.10	1.495 ± 0.041	5.490 × 10^−8^ ± 5.514 × 10^−9^	0.992
20% CLFS	1.35	0.11	1.448 ± 0.037	9.044 × 10^−8^ ± 8.513 × 10^−9^	0.991
7% CBA	1.20	0.26	1.268 ± 0.035	1.575 × 10^−7^ ± 1.647 × 10^−8^	0.985
20% CBA	1.00	0.64	0.985 ± 0.012	1.347 × 10^−6^ ± 1.427 ×10^−7^	0.980
7% CCDW-C	1.49	0.14	1.552 ± 0.049	5.844 × 10^−8^ ± 6.634 × 10^−9^	0.989
20% CCDW-C	1.52	0.10	1.546 ± 0.043	6.752 × 10^−8^ ± 6.838 × 10^−9^	0.991

**Table 4:** Weight loss (%) at different temperature intervals and at depths of 0.5 mm and 22.5 mm.

		Temperature (°C)			
Mortar	Depth (mm)	35–200	200–400	400–500	500–800
OPC	0.5	2.916	1.376	0.908	5.529
	22.5	3.146	1.381	1.134	2.812
20% LFS	0.5	3.623	1.479	0.754	3.451
	22.5	4.110	1.263	1.072	0.665
20% BA	0.5	2.362	1.354	1.509	5.867
	22.5	4.519	1.104	0.888	2.173
20% CDW-C	0.5	2.979	1.237	0.945	4.417
	22.5	5.164	1.364	1.166	1.214
20% CLFS	0.5	3.157	1.889	1.103	4.288
	22.5	4.712	1.380	1.111	1.829
20% CBA	0.5	3.311	1.036	0.938	5.180
	22.5	4.976	1.178	0.979	2.041
20% CCDW-C	0.5	3.570	1.252	0.663	5.050
	22.5	5.065	1.403	1.298	1.336

**Table 5:** Weight loss (%) of Friedel’s salt, monocarboaluminate, portlandite and calcite at depths of 0.5 mm and 22.5 mm.

Mortar	Depth (mm)	% Friedel’s Salt	% Monocarboaluminate	% Portlandite	% Calcite
OPC	0.5	3.085	2.173	3.739	8.031
	22.5	2.844	2.217	4.674	4.559
20% LFS	0.5	3.706	2.954	3.102	5.605
	22.5	2.885	2.557	4.407	0.990
20% BA	0.5	-	-	*	8.386
	22.5	-	-	3.656	4.163
20% CDW-C	0.5	2.929	2.338	3.884	6.353
	22.5	3.093	2.482	4.796	2.121
20% CLFS	0.5	4.027	4.466	4.533	6.644
	22.5	3.094	2.680	4.571	3.189
20% CBA	0.5	-	-	*	7.810
	22.5	-	-	4.027	3.888
20% CCDW-C	0.5	3.442	2.109	2.726	7.742
	22.5	3.178	2.560	5.337	2.348

* Not detected.

## Data Availability

The original contributions presented in this study are included in the article. Further inquiries can be directed to the corresponding authors.
